# Hyperbolic Topological Quantum Sources

**DOI:** 10.1002/advs.202417708

**Published:** 2025-03-17

**Authors:** Lu He, Lei Huang, Weixuan Zhang, Dongning Liu, Huizhen Zhang, Xue Feng, Fang Liu, Kaiyu Cui, Yidong Huang, Wei Zhang, Xiangdong Zhang

**Affiliations:** ^1^ Key Laboratory of advanced optoelectronic quantum architecture and measurements of Ministry of Education Beijing Key Laboratory of Nanophotonics & Ultrafine Optoelectronic Systems School of Physics Beijing Institute of Technology Beijing 100081 China; ^2^ Frontier Science Center for Quantum Information Beijing National Research Center for Information Science and Technology (BNRist) Electronic Engineering Department Tsinghua University Beijing 100084 China; ^3^ Beijing Academy of Quantum Information Sciences Beijing 100193 China

**Keywords:** high utilization efficiency, hyperbolic topological insulator, quantum source, topological protection

## Abstract

Robust Integrable Quantum Optical Sources, Constructed by Topological Boundary States, Are Crucial for the on‐chip Quantum Information Processing. However, Limited by the Bulk‐edge Correspondence, the Implementation of Topological Boundary Channels Necessitates a Substantial Number of Bulk Sites, Which Notably Diminishes the Ratio of Ring Resonators to Generate Quantum Sources. How to Achieve Topologically‐protected Quantum Sources With the Extremely Enhanced Utilization Efficiency of Optical Resonators Remains a Challenge. Here, the First Realization of Hyperbolic Topological Quantum Sources is reported, Which Possess a Dominated Number of Boundary Resonators Than That in the Bulk Domain. Specifically, Hyperbolic Topological Quantum Sources Require Fewer Resources (i.e., the number of ring resonators) to Achieve the Same Level of Brightness Compared With Euclidean Topological Quantum Sources. Furthermore, the Robust Correlated‐ and Entangled‐photon Pairs Are Measured. The Work Possesses Potential Applications in Integrable Quantum Circuits and Suggests a Novel Way on the Exploration of Quantum Physics in Non‐Euclidean Space.

## Graphical abstract

In this work, we present the experimental realization of hyperbolic topological quantum sources on silicon chips, which only require fewer resources to achieve a higher brightness. It shows a high utilization efficiency and a strong robustness.

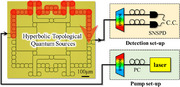

## 1 Introduction

The integrated sources of quantum light are essential components in the development of photonic circuits for on‐chip quantum information processing^[^ ^1^, ^2^, ^3^, ^4^, ^5^, ^6^, ^7^, ^8^, ^9^ ^]^ The stability of their operations directly impacts the overall efficiency of the quantum chip. Most quantum light sources rely on second and third‐order nonlinear processes, such as spontaneous parametric down‐conversion (SPDC)^[^ ^10^, ^11^, ^12^, ^13^, ^14^, ^15^, ^16^, ^17^, ^18^ ^]^ and spontaneous four‐wave mixing (SFWM).^[^ ^19^, ^20^, ^21^, ^22^, ^23^, ^24^, ^25^, ^26^, ^27^ ^]^ However, these on‐chip quantum sources are often susceptible to environmental influences and fabrication errors, making it challenging to obtain robust sources of quantum light. Recent investigations have shown that the combination of topology and quantum optics can offer an alternative approach to address this issue, and numerous intriguing phenomena have been revealed,^[^ ^28^, ^29^, ^30^, ^31^, ^32^, ^33^, ^34^, ^35^, ^36^, ^37^, ^38^, ^39^, ^40^, ^41^, ^42^, ^43^, ^44^, ^45^, ^46^, ^47^, ^48^, ^49^, ^50^, ^51^, ^52^, ^53^, ^54^, ^55^, ^56^, ^57^ ^]^ including the topological quantum‐optics interface,^[^ ^28^ ^]^ topological transport of pre‐produced entangled states,^[^ ^33^, ^36^, ^37^ ^]^ topologically protected cavity quantum electrodynamics,^[^ ^39^ ^]^ and others. Particularly noteworthy is the demonstration of various types of topological sources of quantum light, encompassing topological single‐photon states, correlated biphoton states,^[^ ^30^, ^31^ ^]^ and entangled photon states.^[^ ^32^ ^]^ However, limited by the bulk‐edge correspondence, the *n*th‐order topological phases are always featured by topological boundary states with *n*‐dimensional lower than the bulk that hosts them. Consequently, the volume of topological channels for the generation of quantum light is significantly smaller compared to that of the trivial bulk domain. This limitation hinders the efficiency of utilizing topological photonic chips for quantum source generation, necessitating a larger chip area to achieve an optimal brightness of the quantum source. To realize integrable quantum photonic circuits, it is crucial to maximize the utilization efficiency of nonlinear materials to achieve the optimal performance of quantum sources with a minimal amount of photonic resonators.

On the other hand, the non‐Euclidean geometry pervades the natural world and assumes pivotal roles across diverse domains. Over the past few decades, extensive research has been conducted on hyperbolic lattices,^[^ ^58^, ^59^, ^60^, ^61^, ^62^, ^63^, ^64^, ^65^, ^66^, ^67^, ^68^, ^69^, ^70^, ^71^, ^72^, ^73^, ^74^, ^75^, ^76^, ^77^ ^]^ which correspond to regular tessellations in non‐Euclidean space characterized by a constant negative curvature. Recently, the experimental realization of 2D hyperbolic lattices in circuit quantum electrodynamics,^[^ ^71^ ^]^ topolectrical circuits,^[^ ^73^, ^74^ ^]^ and coupled optical ring resonators^[^ ^75^ ^]^ has sparked a plethora of studies on hyperbolic topological states. One noteworthy characteristic of hyperbolic lattices is that boundary sites consistently occupy a finite fraction of the total sites, irrespective of the structural size. This extraordinary property empowers hyperbolic topological states to showcase a spatial profile predominantly governed by boundary sites. Inspired by the boundary‐dominated hyperbolic topological states, it is intriguing to inquire whether we can achieve hyperbolic quantum sources that are both endowed with topological protection and exhibit remarkable utilization efficiencies.

In this work, we theoretically design and experimentally demonstrate the realization of hyperbolic topological quantum sources on silicon (Si) photonic chips. The constructed hyperbolic topological quantum sources have the capability to generate frequency‐degenerate photon pairs and exhibit high visibility in Hong‐Ou‐Mandel (HOM) interference. Furthermore, we showcase the achievement of time‐energy entanglement using the hyperbolic topological quantum source. The exceptional robustness is also confirmed. As the number of rings increases, the topological quantum source reaches a maximum brightness. In this case, the number of rings required by the hyperbolic quantum source is 1–2 orders of magnitude smaller than that of previously reported topological quantum sources. Our groundbreaking hyperbolic topological quantum source holds immense potential for applications in the field of integratable quantum chips.

## 2 The Theoretical Design of Hyperbolic Topological Quantum Source

**Figure 1:**
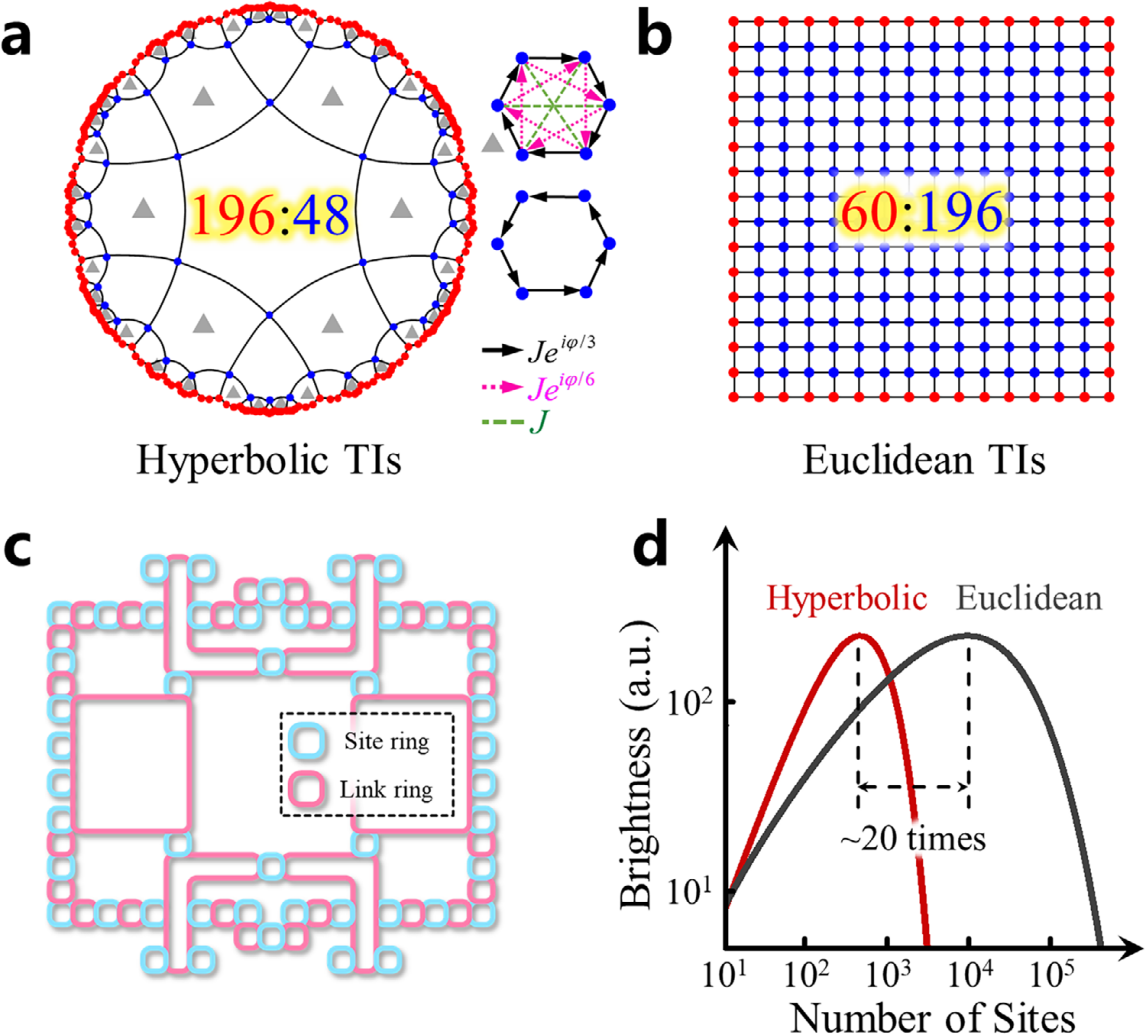
The theoretical design of hyperbolic topological quantum source. a) The illustration of the hyperbolic topological insulators (TIs). The red dots represent the edge sites. Blue dots represent the bulk sites. Black lines represent the coupling. The ratio between bulk and edge sites is 48:196. The right part shows the coupling relationship between sites. Black arrow: the *NN* hopping. Pink arrow: the *NNN* hopping. Green dashed line: the *NNNN* hopping. b) The illustration of the Euclidean topological insulator. The ratio between bulk and edge sites is 196:60. c) The schematic diagram of the designed hyperbolic photonic topological insulators. The pink (blue) ring represents the link (site) ring. d) The brightness of the topological quantum sources is based on the hyperbolic and Euclidean topological insulators.

We start to consider a {6,4} hyperbolic lattice plotted in the Poincaré disk, as shown in **Figure** **1**a. Here, {6,4} is a Schläfli notation, which corresponds to the tessellation of a 2D hyperbolic plane by 6‐sided regular polygons with the coordination number being 4. The bulk and edge lattice sites are represented by blue and red dots, respectively. In order to construct hyperbolic topological edge states, the real and complex‐valued site couplings are suitably designed, as shown in the right chart of Figure 1a. Specifically, the coupling pattern within each hexagon can be classified into two types. Nearly a half number of hexagons (marked by grey triangles) possess the nearest‐neighbor ($J_{1}e^{i\varphi_{1}}$), next‐nearest‐neighbor ($J_{2}e^{i\varphi_{2}}$) and next‐next‐nearest‐neighbor ($J_{3}e^{i\varphi_{3}}$) hoppings, which are represented by the black, pink, and dashed green arrows, respectively. *J_i_* and φ_*i*_ (i = 1,2 and 3) correspond to amplitudes and phases of different hopping strengths. In addition, the remained half of the hexagons only contain the nearest‐neighbor coupling ($J_{1}e^{i\varphi_{1}}$). Based on such a design, our proposed hyperbolic lattice model possesses the non‐trivial real‐space Chern number and can also sustain boundary‐dominated one‐way topological edge states (see Section S1, Supporting Information for details). These characteristics clearly manifest the topological feature of our hyperbolic lattice model.

One of the most important properties of the hyperbolic topological insulator is that the ratio between the amounts of edge and bulk sites is much larger than that of the Euclidean counterpart. For instance, the ratio of edge and bulk sites for the topological insulator existed in {4, 4} Euclidean lattice (Figure 1b) is 60:196 with the total number of lattice sites being 256. Such a value is much smaller than that of our proposed hyperbolic topological insulator (196:48) with nearly the same number of lattice sites. A detailed discussion on the geometric property of hyperbolic lattices is given in Section S2 (Supporting Information). Based on the novel geometry of hyperbolic lattice, if we can utilize the hyperbolic topological insulator to construct a topological quantum source, it is anticipated that a higher utilization efficiency of ring resonators can be achieved compared to its Euclidean counterpart.

For this purpose, we apply the previously constructed hyperbolic photonic topological insulator with coupled ring resonators to realize the hyperbolic topological quantum source with a high utilization efficiency on photonic resonators.^[^ ^75^ ^]^ The schematic diagram of our designed structure is shown in Figure 1c. The photonic ring resonators are classified into two classes, where the blue and pink rings represent the site and linking rings, respectively. In the design process, we can adjust the effective coupling amplitudes *J* (= 17.5 GHz) between two site rings by adjusting the separation distance between site rings and link rings. Additionally, the coupling phase φ_*i*,*j*_ between two site rings can be controlled by manipulating the propagation phase φ_*i*_ within the link ring. Here, all site resonators possess identical geometric parameters, ensuring the same resonant frequency and free spectral range (FSR) of all site resonators. By suitably designing the coupling pattern by each linking ring, our designed coupled ring array can be mapped to the tight‐binding lattice model in Figure 1a, and exhibits hyperbolic topological properties. The detailed design principle and structural parameters can be found in Section S3 (Supporting Information). It is noted that each site ring supports two pseudo‐spin components, which circulate in opposite directions within each site ring resonator. These two pseudospins can form the spin‐up (clockwise) and spin‐down (counterclockwise) topological edge modes, which propagate along the upper and lower boundaries of the hyperbolic structure (see Section S4, Supporting Information for detailed simulation results).

It is worth noting that our designed hyperbolic photonic topological insulator possesses superior performances in realizing quantum optical sources. In the case of Si‐based quantum sources through the SFWM process, the brightness of generated photons relies on both the intensity of pump light in the Si medium, its propagation length, and the losses.^[^ ^24^ ^]^ For the topological lattices, the brightness is related to the length of the edge, that is the number of edge sites. Furthermore, for the coupled ring resonator system, assuming each resonator possesses the same geometrical parameters of the waveguide, the shape of each ring cavity, the Q factor, the material nonlinear coefficient, the power of the pump laser and other parameters, the total brightness can be expressed as a function of the number of ring resonators.^[^ ^24^, ^25^ ^]^ Hence, within a topological system, quantum sources are exclusively generated at edge resonators while bulk resonators make no contribution. Figure 1d presents a comparison between the brightness values for topological quantum sources in Euclidean and hyperbolic spaces as a function of total ring resonator count. It can be observed that the brightness for the hyperbolic topological quantum source (red line in Figure 1d) initially increases with the total number of rings. However, due to the loss in the rings, the brightness reaches its maximum value at a certain total number of rings. If the total number of rings continues to increase, the impact of loss will cause the brightness of the rings to decrease. Correspondingly, the Euclidean topological source also exhibits a similar phenomenon (black line in Figure 1d). However, what differs is that the Euclidean quantum source requires a larger total number of rings to achieve maximum brightness, whose value is ≈20 times greater than the optimal number of rings for the hyperbolic source. In what follows, we explore how to experimentally realize the hyperbolic quantum optical source and measure its nature of the two‐photon interference and the energy‐time entanglement.

## 3 Topologically‐Protected Hyperbolic Quantum Source for Correlated Photon Pair with Non‐Degenerated Frequencies

**Figure 2:**
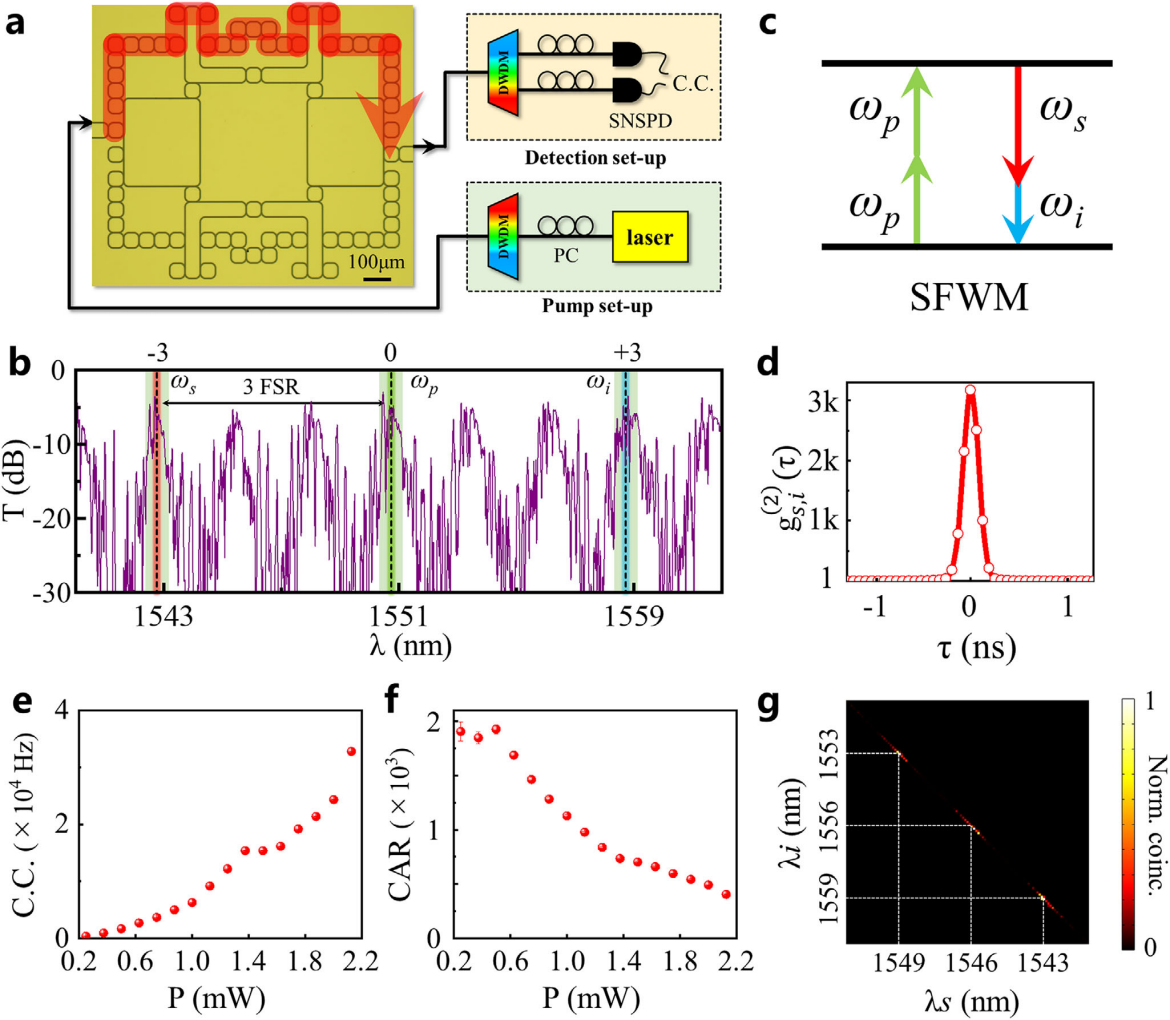
Topologically‐protected hyperbolic quantum source and its characterization. a) The top‐view microscopy image of the hyperbolic topological photonic insulator and the illustration of the experimental set‐up to generate and measure the quantum source. The partial clockwise edge paths are highlighted in red, where the signal and idler photons are generated. PC: polarization controller. DWDM: cascaded dense wavelength division multiplexer. SNSPD: superconducting nanowire single‐photon detector. b) Experimental transmission spectra by exciting the spin‐up mode. The green, orange, and blue dashed lines mark the frequencies of the pump, signal, and idler photons, respectively. c) The schematic diagram of the SFWM process. d) The measured second‐order cross‐correlation function $g_{s,i}^{\left(2\right)}\left(\tau \right)$. e) The C.C. at the output port (adjusted for coupling losses) as a function of the power in the Si waveguide. f) The coincidence to accidental coincidence ratio (CAR) is a function of the power in the Si waveguide. g) The experimental joint‐spectral intensity (JSI) of the signal and idler photons. The wavelength of the pump laser is 1550.92 nm. The wavelength of the signal (idler) photons, λ*s* (λ*i*), is from 1541.35 to 1550.92 nm (from 1551.72 to 1560.61 nm). The C.C. is normalized to maximum. The error bars in (e) and (f) are calculated assuming Poissonian statistics for photon counts.

In this section, we delve into the exploration of the hyperbolic topological quantum source crafted on a 220nm‐thick Si layer atop the silicon‐on‐insulator (SOI) platform (See Experimental Section for detailed procedures). The microscopy image of the fabricated structure is displayed in the left chart of **Figure** **2**a. Remarkably, our fabricated photonic structure aligns with its theoretical design (depicted in Figure 1c).

By injecting a probe laser into this structure and detecting the output light using a power monitor, we are able to experimentally measure the transmission spectrum of the spin‐up pseudospin mode, as represented by the purple line in Figure 2b. During this measurement process, we vary the wavelength of our probe laser spanning from 1540 to 1560 nm, encompassing eight free spectral ranges (FSR) within this interval. Three FSRs have been designated as −3, 0, +3‐order FSRs, respectively. Here, the FSR of our structure is ≈330 GHz. It is shown that the large‐valued transmissivities appear within each FSR (as highlighted by green regions), and the corresponding frequency ranges are also matched to eigenfrequencies of hyperbolic topological edge states. In this case, the periodic high‐transmission regions manifest the excitation of hyperbolic topological edge states, where the light can propagate along the uppermost edge of our structure (as indicated by the red arrow in Figure 2a). The low transmittivities for the remained frequency domains are related to the trivial bulk states. The experimental transmission spectrum possesses a good consistency with the simulation result (see Section S4, Supporting Information). It is noted that the periodic edge bands can be employed to generate the quantum optical source. Additionally, we find that the lattice spectra in the different FSRs do not seem to be replicas of each other. That is because the usage of the big link rings introduces some interferences in the total transmission spectra (see Section S3.3, Supporting Information).

In the following, we show the biphoton emitting assisted by the above fabricated hyperbolic topological insulator. Here, the homemade pump and detection set‐ups, which are illustrated by two insets in Figure 2a, are used. The continuous‐wave mono‐color pump light at ω*_p_* is injected into the experimental system, where the cascaded dense wavelength division multiplexers (DWDMs) are used to remove noise photons at other frequencies (ω ≠ ω*_p_*) and the polarization controllers (PCs) can adjust the polarization to achieve the maximum grating coupling efficiency. The input light is suitably designed to solely excite the spin‐up pseudo‐spin mode, where the hyperbolic topological edge states propagating along the clockwise direction can be excited (shown by the red arrow). The hyperbolic topological boundary state can trigger the appearance of the signal (at ω*_s_*) and idler (at ω*_i_*) photons induced by SFWM at upper‐edge resonators, where 22 on‐site ring resonators are used. The law of energy conservation ensures 2ω*_p_* = ω*_s_* + ω*_i_*, as shown in Figure 2c. Additionally, the momentum matching can also be satisfied in the SFWM process due to the linear dispersion of the hyperbolic edge state (see Section S5, Supporting Information for details).

In experiments, we set the input wavelength being 1550.92 nm (equal to 2π/ω*_p_*, and marked by the green line in Figure 2b), and two wavelengths of signal and idler photons are 1542.94 nm (2π/ω*_s_*, and marked by the orange line) and 1558.98 nm (2π/ω*_i_*, and marked by the blue line), respectively. Then, the generated photons are transferred into the optical fiber through the 1D grating, where another DWDM is used for filtering out the signal and idler photons toward different channels. Finally, we employ the fiber‐coupled superconducting nanowire single‐photon detector (SNSPD) to detect the coincidence counts (C.C.) of the signal and idler photons. The C.C. is executed and the coincidence time window is taken as 64 ps in experiments.

Figure 2d shows the measured second‐order cross‐correlation function,^[^ ^30^ ^]^ $g_{s,i}^{\left(2\right)}\left(\tau \right)$, which is the normalized probability of detecting signal and idler photons separated by time τ. For two uncorrelated sources, *g* ^(2)^= 1. In our hyperbolic quantum source, we get the maximum $g_{s,i}^{\left(2\right)}\approx 3176$ at τ = 0. Here, the pump power of the photonic chip is P_in_ = 1 .4 mW. It is shown that the measured $g_{s,i}^{\left(2\right)}$ can be perfectly fitted by a Gaussian function with the full‐width half maximum (FWHM) being 180 ps. The appearance of a coincidence counting indicates that the generated signal and idler photons are indeed correlated with each other, where a signal (or idler) photon is detected in one channel, and an idler (or signal) photon is destined to be detected in the other channel. Additionally, the C.C. of our hyperbolic quantum source approximately increases as the square of the input power, as shown in Figure 2e. We note that the maximum value of C.C. is 4 × 10^6^ with P_in_ = 2.1 mW in 120 s, and the associated C.C. rate is ≈3.3 × 10^4^ Hz. The signal‐to‐noise ratio of our hyperbolic topological source can be evaluated by the coincidences‐to‐accidentals ratio (CAR), which equals the ratio between the average value of C.C. within the FWHM and that in the range far away from the fitted by a Gaussian profile. Figure 2f displays the measured CAR as a function of the pump power. We can see that the maximum value of CAR is ≈1927. As far as we know, we measure the highest CAR of topological quantum sources to date. In addition, we also note that the CAR decreases inversely with the pump power, which meets the expectation of the SFWM process.^[^ ^24^, ^25^, ^78^ ^]^ In this case, we can see that our hyperbolic topological quantum source can exhibit a good performance both in the quantum brightness and CAR. Additionally, the other frequency channels in the communication band can also be used to generate the correlated biphoton. These results indicate that our hyperbolic quantum source possesses a good performance in the working bandwidth (see Section S6, Supporting Information for details). The experimental results of the $g_{s,i}^{\left(2\right)}$ mainly rely on the CAR of the quantum source and the ability of test systems to filter out the pump photons. For these two elements, our experiments show the better effect. For example, the filters used in the experiments have a high extinction ratio (up to 120 dB). Thus, we measured a high value of the $g_{s,i}^{\left(2\right)}$.

Furthermore, we measure the joint‐spectral intensity (JSI) at a fixed wavelength of the pump light at 1550.92 nm to study the spectral correlation of signal and idler photons, as shown in Figure 2g (see Section S7.1, Supporting Information the detailed experimental set‐ups). Here, the signal and idler photons are filtered out in the wavelength range matching from the −3‐ to +3‐order FSRs. There are three sets of nonzero JSIs, which correspond to three cases with the signal and idler photons being located within −1‐, +1‐order FSRs, −2‐, +2‐order FSRs, and −3‐, +3‐order FSRs. Such a phenomenon is consistent with the requirement of energy conservation in the process of SFWM, where the signal and idler photons with non‐zero valued JSIs must be located in two different FSRs with the same distance to the 0‐order FSR for the pump light.

In addition, it is worth noting that the value of JSIs is nearly zero within the bulk‐mode frequency range even though the law of energy conservation is satisfied. We think that there are two possible reasons. The first one is that in the frequency range of the bulk mode, the transmission is very low. Even if signal and idler photons are generated, these photons cannot be output from the output port. Second, the dispersion of the edge state is linear, which can satisfy the phase‐matching condition of the SFWM. The bulk dispersion is scattered, which does not meet the phase‐matching condition of the SFWM (see Section S5, Supporting Information). Therefore, it is impossible to efficiently generate two‐photon pairs in the bulk mode. Therefore, the JSI of the measured bulk mode is 0.

## 4 Experimental Demonstration of the Robustness of the Hyperbolic Quantum Source

**Figure 3:**
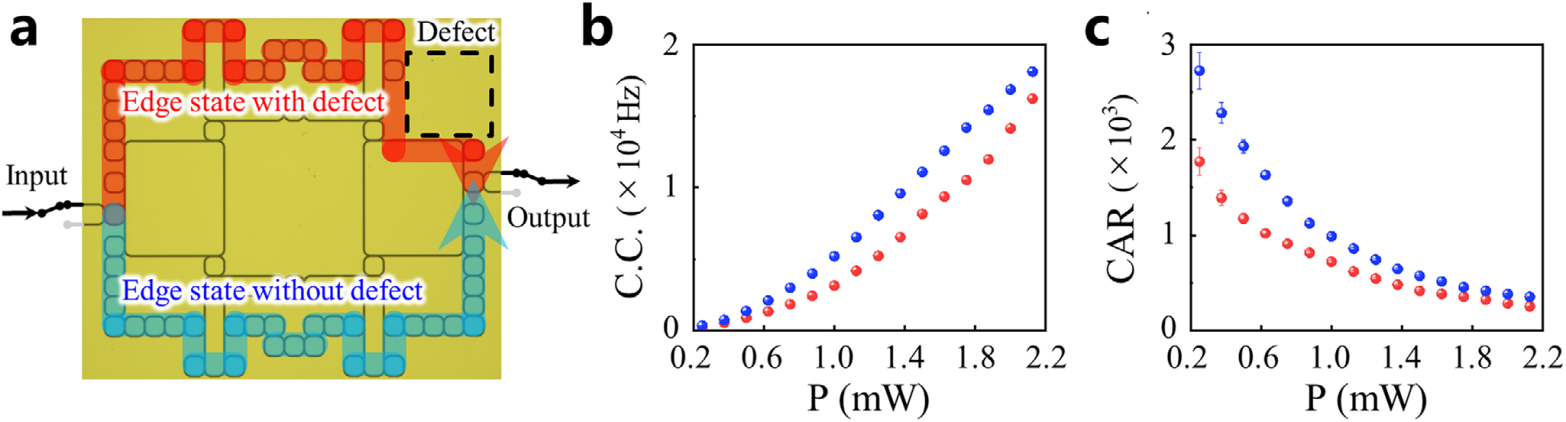
The experimental demonstration of the robustness of the hyperbolic topological quantum source. a) The top‐view microscopy image of the hyperbolic photonic topological insulators with the defect. b) The C.C. at the output port (adjusted for coupling losses) as a function of the power in the Si waveguide. c) The CAR is a function of the power in the Si waveguide. The error bars in (b) and (c) are calculated assuming Poissonian statistics for photon counts.

In order to validate the robustness of the hyperbolic topological quantum source, we also fabricate a sample with a defect, as illustrated in **Figure** **3**a. The imperfection is introduced by selectively removing a set of rings within the hyperbolic structure. Under this specific defect configuration, it is anticipated that the signal and idler photons are generated along the upper optical path (highlighted by the red arrow), circumventing the defect assisted by topological boundary states.

In experiments, we employ the same experimental setup (as depicted in Figure 2a) to measure the C.C. of correlated biphoton states generated by the defective hyperbolic topological quantum source. The experimental result illustrating the relationship between C.C. and pump light power is presented in the red dots of Figure 3b. Notably, C.C. approximately increases quadratically with input power. Remarkably, in order to be compared with the case without any defects, we carried out an additional experiment to measure the C.C. of correlated biphoton states generated along the lower optical path (highlighted by the blue arrow). By comparison, the brightness of our hyperbolic topological quantum source remains unaffected at a consistent level, demonstrating its robustness against defects. Additionally, we investigate variations in CAR as a function of input power, as shown in Figure 3c. Strikingly, even with defects present in our hyperbolic topological quantum source, it still achieves high CAR values (>1800). Consequently, we can conclude that our hyperbolic topological quantum source exhibits remarkable resilience against defects. These experimental findings align well with theoretical analysis (see Section S8, Supporting Information for details).

Additionally, we also perform the robustness comparison of the hyperbolic, square Euclidean, and rectangular Euclidean photonic topological insulators against the random disorder, the simulation results show that the hyperbolic topological insulators have the same degree of topological robustness as the square Euclidean topological insulators. As for the rectangular Euclidean topological insulators, even if it has a large ratio between edge sites and bulk sites, its topological robustness is weaker than that of a hyperbolic or square Euclidean topological insulators (see Section S9, Supporting Information for details).

## 5 Hong‐Ou‐Mandel Interference of the Hyperbolic Topological Quantum Source

**Figure 4:**
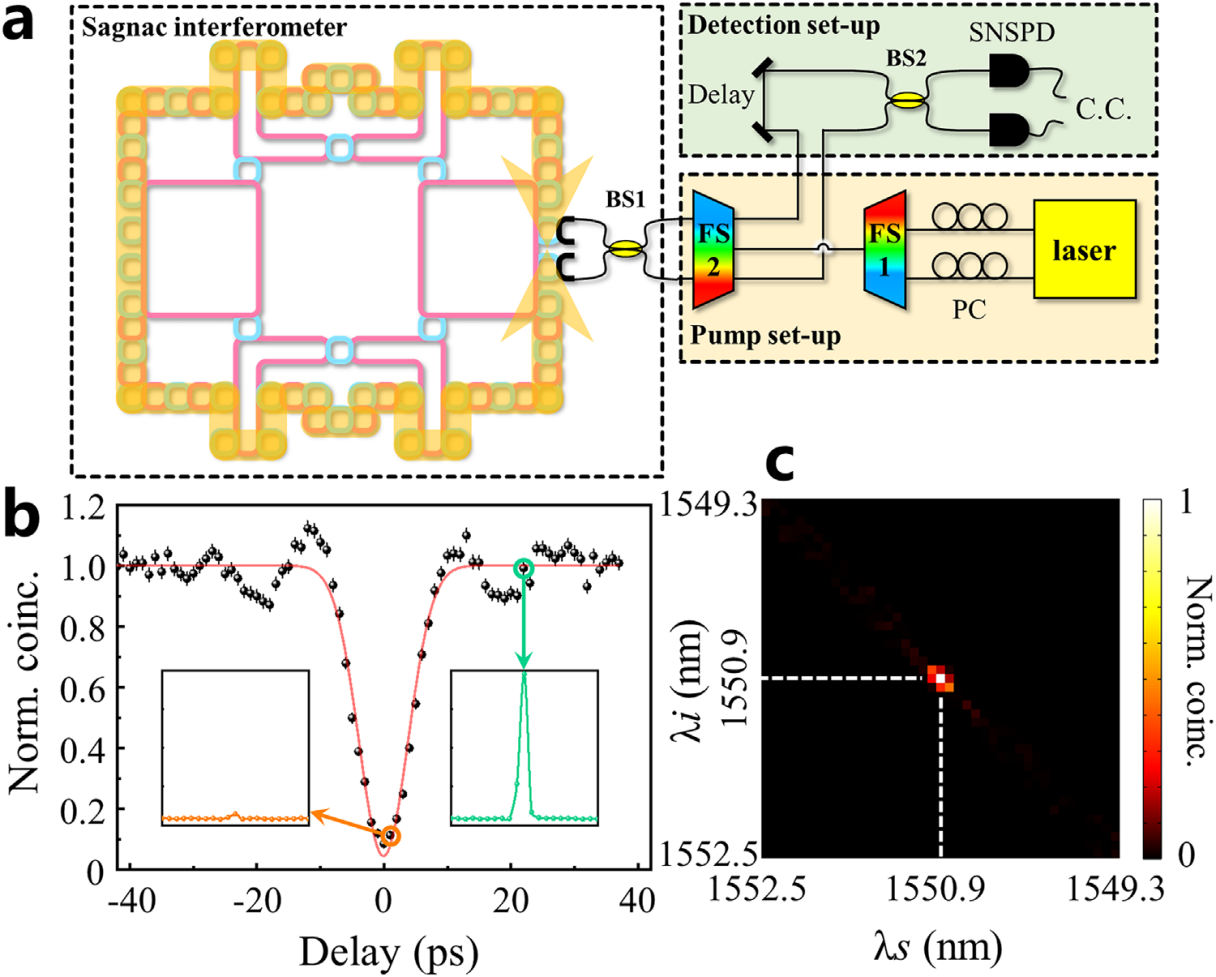
Experimental demonstration of the HOM interference. a) The experimental set‐up, includes a Sagnac loop for generating the indistinguishable photon pairs. b) The experimental results of the HOM interference. The visibility is ≈95.6%. Black dots: Normalized C.C. Red line: The fitted curve by Gaussian function. The error bars are calculated assuming Poissonian statistics for photon counts. The inset: the time‐resolved coincidences between the two frequency‐degenerate photons when the delay is set in and off the HOM dip, corresponding to the green and orange circles, respectively. c) The experimental JSI of the indistinguishable signal and idler photons. The wavelengths of the pump laser are 1542.94 and 1558.98 nm. The wavelength of the signal (idler) photons, λ*s* (λ*i*), is from 1549.3 to 1552.5 nm. The C.C. is normalized to maximum.

Except for the generation of correlated photon pairs with different frequencies, in this part, we focus on the realization of quantum interferences of two photons based on the hyperbolic photonic topological insulator. To generate such a frequency‐degenerate biphoton state, we build a Sagnac interferometer,^[^ ^19^, ^31^ ^]^ which consists of the hyperbolic photonic topological insulator and the first 50:50 beam splitter (BS1), as shown in the left part of **Figure** **4**a. The pump and detection set‐ups are presented in the right chart. Here, a pair of continuous‐wave mono‐color pump lights with corresponding wavelengths being 1542.94 and 1558.98 nm (matched to −3 and +3 FSRs) are combined by the first filter system (FS1) and injected into the Sagnac loop. After going through the second filter system (FS2), both the two lights can be coupled into the top and bottom ports by the BS1. See Methods for the detailed functions of the FS1 and FS2. In this case, the spin‐up and spin‐down topological edge modes are simultaneously excited by each pump light. Due to the dual‐pump SFWM process, two frequency‐degenerate and path‐entangled photons (at the wavelength being 1550.92 nm) are expected to be generated along the clockwise or counterclockwise paths, which are expressed as |2〉_*CW*_|0〉_*CCW*_ and |0〉_*CW*_|2〉_*CCW*_, respectively. When these two biphoton states go around the hyperbolic structure, they re‐meet at the BS1 and interfere with each other, triggering the appearance of the anti‐bunching state (|1〉_*top*_|1〉_*bottom*_) with two photons separately coupled to top‐ and bottom‐right ports of the BS1. Such an anti‐bunching state possesses two frequency‐degenerate photons. We note that the Sagnac interferometer possesses the ability to self‐stabilization the phase so that the additional phase modulation is unnecessary. Then, the generated biphoton state |1〉_*top*_|1〉_*bottom*_ is injected into the detection setup (the green box), where the second 50:50 coupler (BS2) is applied to test the HOM interference effect. Here, a tunable delay line in a single path is used to control the time difference of two photons arriving at the BS2.

The experimental results of the HOM interference with different delay times are presented in Figure 4b. It is clearly shown that a significant dip of C.C. appears with the delay time being zero, manifesting the appearance of HOM interference of two photons. To further illustrate the C.C. results, we plot two C.C. histograms with the delay time being 0 ps (enclosed by the orange circle in Figure 4b) and 20 ps (enclosed by the green circle in Figure 4b), as shown in the inset of Figure 4b. We can see that the value of the C.C. peak is significantly decreased (shown in the left inset) when two photons simultaneously arrive at the BS2. While, as for the case of the arriving times of photons being staggered, the coincidence counting peak reappears (shown in the right inset). These results clearly indicate that the HOM interference indeed happens at the BS2. And, a high visibility of 95.6% is also obtained, which demonstrates the indistinguishable property of the generated biphoton based on the hyperbolic topological quantum source.

Additionally, we also measure the JSI for the indistinguishable photons, as shown in Figure 4c (see Section S7.2, Supporting Information for the detailed experimental set‐ups). Here, the signal and idler photons are indistinguishable in frequencies. The JSI image further demonstrates that the hyperbolic topological quantum source possesses a good property to generate the indistinguishable biphoton.

## 6 Energy‐Time Entanglement of the Hyperbolic Topological Quantum Source

**Figure 5:**
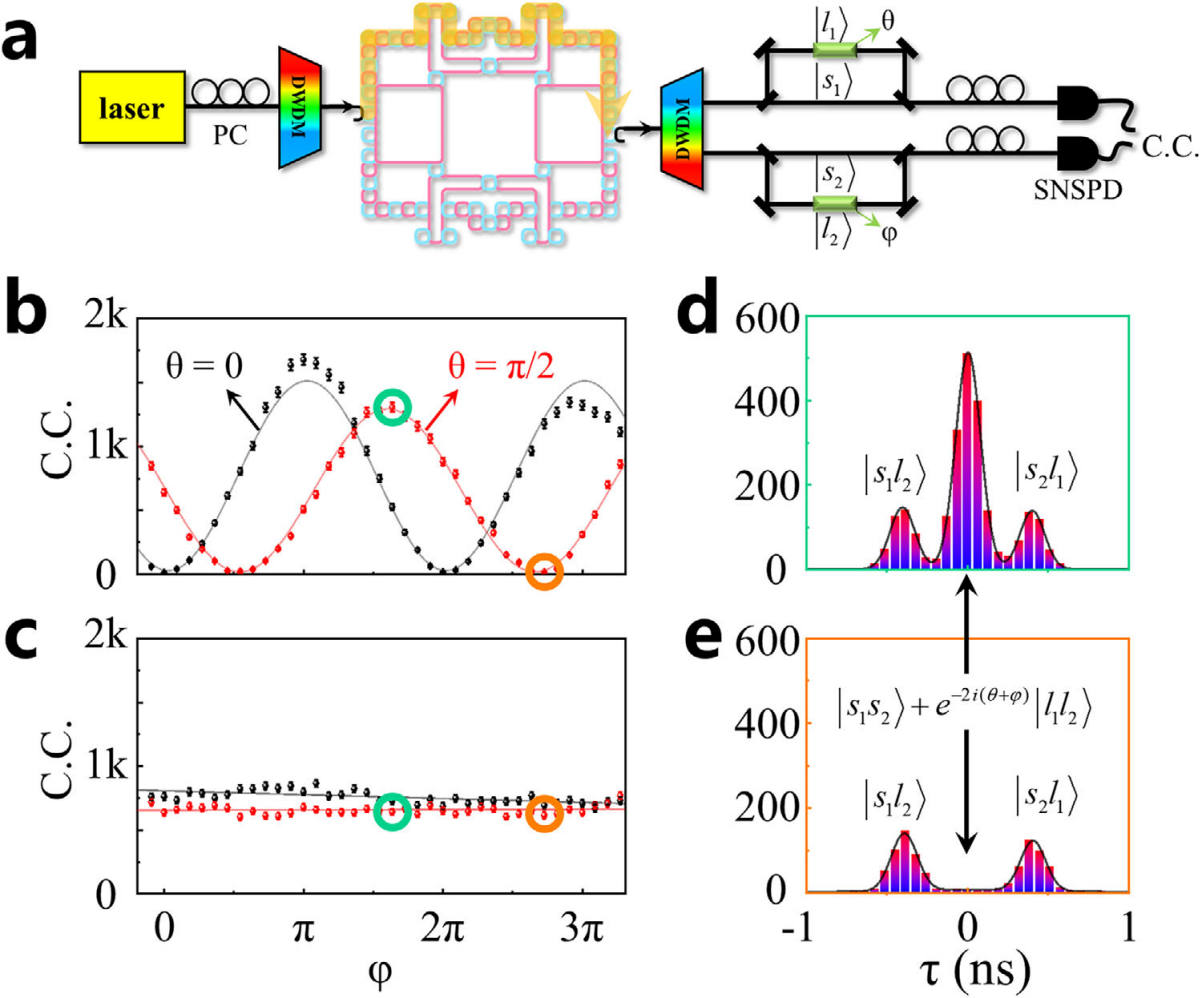
Energy‐time entanglement of the hyperbolic topological quantum source. a) The schematic of the experimental setup with the Franson interferometer demonstrates the energy‐time entanglement of the hyperbolic topological quantum source. The delay between the short and the long paths is ≈400 ps. b,c) The experimental results of two‐photon interferences under two nonorthogonal bases. (b) corresponds to the C.C. of the central peak. The visibilities of the black and red curves are 98.38 ± 0.39% and 97.60 ± 0.49%, respectively. (c) corresponds to the sum of the C.C. of left and right peaks. The error bars in (b) and (c) are calculated assuming Poissonian statistics for photon counts. d,e). Experimental histograms of coincidence counting at two points marked in green and orange circles in (b) and (c). The black lines are the fitting curves by the sum of three Gaussian functions.

Beyond the generation of correlated two‐photon states, in this part, we show that our hyperbolic topological quantum source can also generate the energy‐time entangled biphoton states. Similar to the case for the generation of correlated two photons with different frequencies, the single‐wavelength pump light is also applied to inject into the sample, and solely excite the spin‐up topological edge state, as shown in **Figure** **5**a. Then, two correlated photons are coupled away from the hyperbolic photonic topological insulator and enter into the right DWDM, where the two photons are filtered and divided into two paths. To measure the entanglement of these two photons, two Franson interferometers are constructed, where a time delay of 400 ps between the long (containing a phase modulator, marked by green blocks) and short paths is applied. At the long path, the employed phase modulators can add the additional phases of θ and φ. Finally, two SNSPDs are used to detect the C.C.

There are four possible two‐photon states after the Franson interference process, being expressed as |*s*_1_ *s*_2_〉, |*l*_1_ *s*_2_〉, |*s*_1_ *l*_2_〉, and |*l*_1_ *l*_2_〉. For example, the two‐photon state |*s*_1_ *s*_2_〉 corresponds to the case with the first and second photons going through the top‐ and bottom‐short paths, respectively. It is noted that two biphoton states |*s*_1_ *s*_2_〉 and |*l*_1_ *l*_2_〉 processes at the same time delay two photons, making these two states indistinguishable through the C.C. measurement. By incorporating two extra phases θ and φ along two long paths, these two indistinguishable states can be expressed as |*s*_1_ *s*_2_〉 + *e* ^−2*i*(θ + φ)^|*l*_1_ *l*_2_〉. We use the optical fiber interferometer to realize the phase modulation. The electro‐optical effect of the electrically modulated phase shifter is used to realize the phase modulation. In this case, the interference fringes can be obtained by adjusting the phase value φ from 0 to 4π with θ being fixed at 0 (the black line) or π/2 (the red line), as shown in Figure 5b. Differently, the biphoton states |*s*_1_ *l*_2_〉 |*l*_1_ *s*_2_〉 possess the non‐zero time delays of two photons, making these two states distinguishable in the time domain. In this case, there is no interference fringe related to these two states by tuning the value of φ, as shown in Figure 5c. To further illustrate the difference between indistinguishable and distinguishable two‐photon states, we show the experimental histograms of C.C. with θ = π/2 and φ = 2π (marked by green circles), and θ = π/2 and φ = 3π, (marked by orange circles), as shown in Figure 5d,e. As for the case with θ = π/2, φ = 2π, the middle, left, and right peaks correspond to two‐photon states of |*s*_1_ *s*_2_〉 + *e* ^−2*i*(θ + φ)^|*l*_1_ *l*_2_〉, |*s*_1_ *l*_2_〉, and |*l*_1_ *s*_2_〉, respectively. The maximum value for the middle C.C. peak originates from the constructive interference for the indistinguishable two‐photo state. While, such a peak disappearance under a destructive interference with θ = π/2 and φ = 3π. In addition, the left and right peaks related to a pair of distinguishable two‐photo states are always unchanged, being consistent with the non‐entanglement property of these two states of |*s*_1_ *l*_2_〉, and |*l*_1_ *s*_2_〉.

Finally, the calculated visibilities of the black and red fitted curves, which can be defined as *v* = (C_max_‐C_min_)/(C_max_+C_min_) with C_max_ and C_min_ corresponding to the maximum and minimum values of the measured C.C. rates, equal to 98.38 ± 0.39% and 97.60 ± 0.49% (>70.7%), respectively. These are the raw visibilities. We also perform 10 times additional measurements and a long‐coincidence‐counting‐time measurement of the energy‐time entanglement curves (see Section S10, Supporting Information for these experimental results). All the experimental results prove that our hyperbolic topological quantum source can generate two energy‐time entangled photons with high visibilities. It is worth noting that the measured visibilities of our hyperbolic topological quantum source are larger than that of the Euclidean topological quantum sources ≈92%.^[^ ^31^ ^]^ Two photons with the interference visibility being larger than 98% may be used in the field of quantum key distribution to reduce bit error rates.^[^ ^79^ ^]^ These experimental results indicate that our hyperbolic topological quantum source can generate energy‐time entangled biphotons.

## 7 Discussion

In summary, we have successfully designed and fabricated hyperbolic topological quantum sources utilizing coupled ring resonators on the silicon photonic chip. Through theoretical modeling and experimental validation, we have confirmed that hyperbolic topological quantum sources require fewer resources (i.e., the number of ring resonators) to achieve the same level of brightness compared with Euclidean topological quantum sources. Moreover, we have observed both HOM interference and time‐energy entanglement of the generated photons by the hyperbolic quantum source, showcasing the exceptional efficiency of our device in capturing these intricate quantum phenomena. Furthermore, the robustness of our hyperbolic topological quantum source has been demonstrated. In the future, by further optimizing the chip space utilization, hyperbolic quantum sources using fewer rings are expected to achieve smaller footprints than Euclidean quantum sources. This groundbreaking work introduces innovative designs for on‐chip integrated quantum optical sources that hold promise in addressing the challenge of generating photon sources with strong robustness and high utilization efficiency.

## 8 Experimental Section

### Measurement Method

The continuous wave laser (1500–1630 nm) was employed to measure the sample in the experiment. The incident light was first coupled to the single‐mode fiber (SMF). Then the polarization controllers were used to adjust the polarization state of the light. Lights were entered into the chip by the fiber array. The output signals were collected by another SMF of the fiber array and detected by a high‐speed optical power monitor. To sweep the wavelength of the laser, the transmission spectrum in the whole near‐infrared band was obtained. The insertion loss of the sample was ≈5 dB.

To generate the biphoton, the continuous wave laser (key sight N7714A) was employed to pump the light with the wavelength being 1550.92 nm in the experiment. The incident laser was first coupled to the SMF, combined with the DWDMs, and injected into the chip by the fiber array. The generated photons from the chip coupled to the SMFs of the fiber array, went through the DWDMs (filter out the pump light), and were counted by the SNSPDs. The efficiencies of single photon detectors were ≈85% and dark count rates were ≈100 Hz. Finally, a coincidence measurement between the two photons was performed. The impact of time jitter on coincidence counting was considered. The jitter of the SNSPDs was ≈80 ps, this was indeed larger than the coincidence time window set. The FWHM of our coincidence count peak was 200 ps (≈4 coincidence time windows). Therefore, 4 bins were used to process the data of the coincidence counting, and it can cover the entire match peak.

The average power of the CW laser was adjusted in the range from 4 to 34 mW. The power of 1.4 mW was the power in the waveguide, which corresponds to the average power of the CW laser was 22 mW. This value (1.4 mW) was adjusted by the coupling loss. The coupling loss consists of three parts, including the fiber system loss, the loss of the filter (cascaded DWDM), and the input grating loss. Their values were 3, 2, and 7 dB, respectively. In our estimation, if the pump power was more than 4 mW (0.25 mW in the waveguide), the high‐CAR biphoton generation can be observed.

To realize the FS1 and FS2 in Figure 4a, two and four DWDMs were employed. The connection type can be found in Section S11 (Supporting Information). Specifically, the FS1 achieved the function of combining two beams of light with different wavelengths (1542.94 and 1558.98 nm) together and injecting them into the FS2. The function of the FS2 was to filter the mixed light into two left output ports (connecting to the BS1, shown in Figure 4a). The light of the wavelength being 1542.94 nm (1558.98 nm) went to the left‐upper (left‐lower) port. Additionally, after going through the Sagnac interferometer, the pump lights and the generated photons (1550.92 nm) came back to the left ports of the FS2. Finally, the FS2 could also filter out generated photons and make them enter into the detection set‐up.

### Sample Fabrication

The samples were fabricated using standard complementary metal‐oxide‐semiconductor processes and 248 nm deep ultraviolet (DUV) lithography processes. The substrate was a silicon‐on‐insulator wafer with a 220‐nm‐thick top Si layer. First, a thin oxide layer was formed on the wafer by thermal oxidation. The wafer was coated with a positive photoresist. After several DUV lithography and inductively coupled plasma etching processes, a 450 nm strip waveguide with 220 nm thickness and the coupling grating structure with polycrystalline silicon were fabricated. The coupling gap was 247.5 nm. The etching depth for the sample was 220 nm. A special annealing process was performed to smooth the sidewall of the device. Subsequently, to maintain the up‐down symmetry of the structure, a layer of 1‐µm‐thick cladding oxide was deposited by plasma‐enhanced chemical vapor deposition.

## Conflict of Interest

The authors declare no conflict of interest.

## Author Contributions

Lu He, Lei Huang, and Weixuan Zhang contributed equally to this work. Lei Huang and Weixuan Zhang finished the theoretical design of the hyperbolic topological structure. Lu He finished the theoretical model of the hyperbolic topological quantum source and designed the experiments. Lu He finished experiments with the help of D.L., H.Z., X.F., F.L., K.C. under the supervision of Wei Zhang and Y.D.H., Lu He, Weixuan Zhang, and X.D.Z. wrote the manuscript. X.D.Z. initiated and designed this research project.

## Supporting information



Supporting Information

## Data Availability

The data that support the findings of this study are available from the corresponding author upon reasonable request.
