# Supplementary material for: Hyperbolic Topological Quantum Sources
Source: Adv Sci (Weinh). 2025 Mar 17;12(18):2417708. doi: 10.1002/advs.202417708 (PMC12079522; doi:10.1002/advs.202417708)
Supplement: Supplementary file 1 — Supporting Information [file ADVS-12-2417708-s001.pdf]

ADVANCED SCIENCE
Open Access

Supporting Information

for *Adv. Sci.*, DOI 10.1002/advs.202417708

Hyperbolic Topological Quantum Sources

Lu He, Lei Huang, Weixuan Zhang, Dongning Liu, Huizhen Zhang, Xue Feng, Fang Liu, Kaiyu Cui, Yidong Huang, Wei Zhang and Xiangdong Zhang**

Supplementary Materials for

Hyperbolic topological quantum sources

Lu He[1*], Lei Huang[1*], Weixuan Zhang[1*], Dongning Liu[2], Huizhen Zhang[1], Xue Feng[2], Fang Liu[2], Kaiyu Cui[2], Yidong Huang[2,3], Wei Zhang[2,3+] and Xiangdong Zhang[1$]

[1]Key Laboratory of advanced optoelectronic quantum architecture and measurements of Ministry of Education, Beijing Key Laboratory of Nanophotonics & Ultrafine Optoelectronic Systems, School of Physics, Beijing Institute of Technology, 100081 Beijing, China.

[2]Frontier Science Center for Quantum Information, Beijing National Research Center for Information Science and Technology (BNRist), Electronic Engineering Department, Tsinghua University, Beijing 100084, China

[3]Beijing Academy of Quantum Information Sciences, Beijing 100193, China.

**These authors contributed equally to this work. [$+]Author to whom any correspondence should be addressed: zhangxd@bit.edu.cn; zwei@tsinghua.edu.cn*

S1. Analysis of the tight-binding models of hyperbolic topological lattices.

We consider a {6, 4} hyperbolic lattice in the Poincaré disk, where the center of a hexagon locates at the origin, as shown in Fig. 1(a) in the main text. We call this lattice model as the face-centered hyperbolic lattice. The coupling patterns inside all hexagons can be divided into two categories. Nearly a half number of hexagons possess the nearest-neighbor (*NN*) hopping of $Je^{i\varphi/3}$, the next-nearest-neighbor (*NNN*) hopping of $Je^{i\varphi/6}$ and the next-next-nearest-neighbor (*NNNN*) hoppings of J. The remained half of hexagons only contain the *NN* coupling of $Je^{i\varphi/3}$. In this case, the hyperbolic lattice model can be effectively described by a tight-binding Hamiltonian as:

$$\hat{H} = \sum_i \omega_0 a_i^\dagger a_i + \sum_{<i,j>} Je^{i\varphi/3} a_i^\dagger a_j + \sum_{<<i,j>>} Je^{i\varphi/6} a_i^\dagger a_j + \sum_{<<<i,j>>>} J a_i^\dagger a_j + h.c.. \tag{S1}$$

with $a_i^\dagger(a_i)$ being the creation (annihilation) operator at site i. ω_0 is the on-site potential of each lattice site. The bracket <···> indicates the summation being restricted within all *NN* sites of the *i*th site. Other two brackets <<···>> and <<<···>>> correspond to summations being restricted within a half number of *NNN* and *NNNN* sites of the *i*th site.

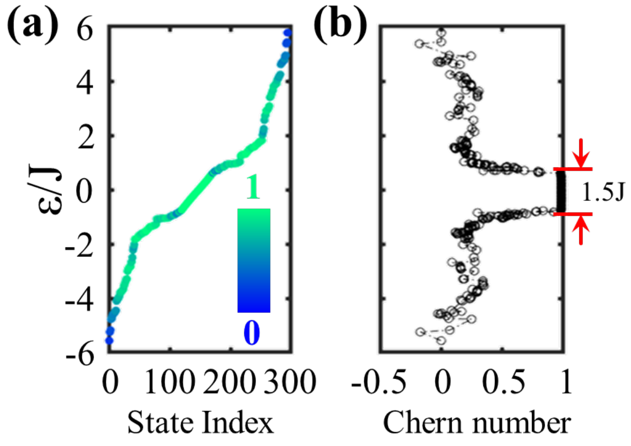

Fig. S1. Analysis of the tight-binding models of hyperbolic topological lattices. (a). The eigenspectra of the three-layer face-centered hyperbolic model. (b). The real-space Chern numbers of the three-layer face-centered hyperbolic model**.** Here, we set the coupling phase φ to π.

It is noted that the complex-valued *NNN* couplings can create the staggered flux into a half number of hexagons, that can break the time-reversal symmetry of the system and introduce non-trivial topologies. The calculated eigenspectra (ε) of the three-layer hyperbolic model with φ equaling to π are presented in two left charts of Figs. S1(a). Other parameters are set as $J = 1$ and $\omega_0 = 0$. The color map represents the localization strength of eigenstates on lattice sites at the third layer, that is quantized by $V(\varepsilon_n) = \sum_{i\in L=3} |\boldsymbol{\phi}_i(\varepsilon_n)|^2 / \sum_{i\in L=[1,3]} |\boldsymbol{\phi}_i(\varepsilon_n)|^2$ with $\boldsymbol{\phi}_i(\varepsilon_n)$ being the eigenmode at $\varepsilon = \varepsilon_n$. It is shown that there are a large number of eigenstates exhibiting boundary-localized spatial profiles. To further determine the topological properties of these edge states, we calculate the corresponding real-space Chern numbers shown in Fig. S1(b). It is shown that the non-zero platform of the real-space Chern number appears around the eigenenergy of $\varepsilon = 0$ with $\varphi = \pi$, indicating the existence of Chern-class topological edge states in our designed hyperbolic lattices. It is worth noting that the calculated real-space Chern number is much closer to one than that of previously proposed {6,4} hyperbolic Haldane model with the same number of lattice sites [S1], showing a good superiority of our designed hyperbolic topological lattice model. These results clearly show that non-trivial topological edge states exist in our designed face-centered hyperbolic topological lattice with suitably engineered staggered flux.

S2. The detailed relationship between the numbers of bulk and edge sites of hyperbolic lattices.

Here, we use the Schläfli notation $\{p, q\}$ to describe the hyperbolic lattice, where p represents that the unit cell of a 2D hyperbolic lattice is p-sided regular polygons, and q represents the coordination number of every lattice site. Now let's derive the relationship between the numbers of bulk and edge sites of hyperbolic lattices

Fig. S2 shows a 3-layer hyperbolic lattice with p=6 and q=4. The red, blue, and pink sites represent the lattice sites in the 1st, 2nd, and 3rd layers. The gray triangles mark the coupling relationship, which is mentioned in the main text. For the lattice sites of each layer, there are two different cases. The first one is that the lattice site is not directly connected to the previous layer, we name it as **a** lattice sites (highlighted by the red square). and the second one is the lattice site that is directly connected to the previous layer, we name it the **b** lattice site (highlighted by blue square). We use the symbol of a_i to represent the number of **a** lattice sites in the ith layer, and b_i to represent the number of **b** lattice sites in the ith layer.

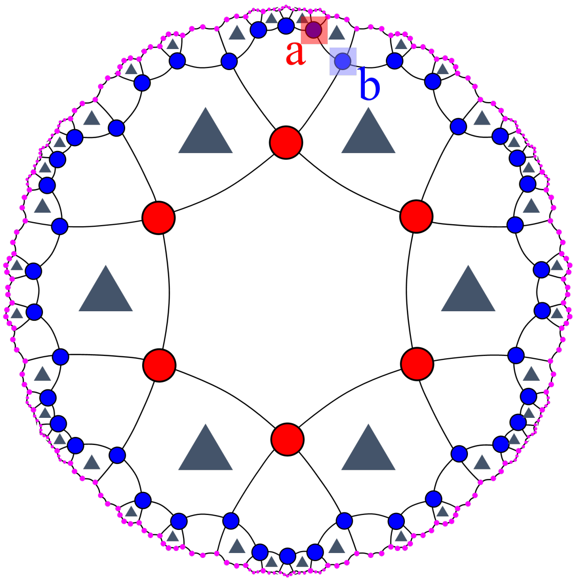

Fig. S2. The schematic diagram of the 3-layer hyperbolic lattices.

Now, let us deduce the relationship between a$_i$ and b$_i$ in multi-layer hyperbolic lattices. The connection relationship between **a** lattice site in a certain layer and the lattice sites in the next layer is shown in Fig. S3(a). We can see that the number of **a** lattice sites of the next layer connected to **a** lattice sites in this layer is $(p-3)(q-3)+p-4$, and the number of **b** lattice sites of the next layer connected to **a** lattice sites in this layer is $q-2$. Fig. S3(b) shows that the connection relationship between **b** lattice site in a certain layer and the lattice sites in the next layer. In same way, the number of **b** lattice sites of the next layer connected to **b** lattice sites in this layer is $(p-3)(q-4)+p-4$, and the number of **b** lattice sites of the next layer connected to **b** lattice sites in this layer is $q-3$.

Thus, the relationship between a$_i$, b$_i$ and a$_{i+1}$, b$_{i+1}$ can be written as:

$$a_{i+1} = a_i\big((p-3)(q-3)+p-4\big) + b_i\big((p-3)(q-4)+p-4\big)$$
$$b_{i+1} = a_i(q-2) + b_i(q-3) \tag{S2}$$

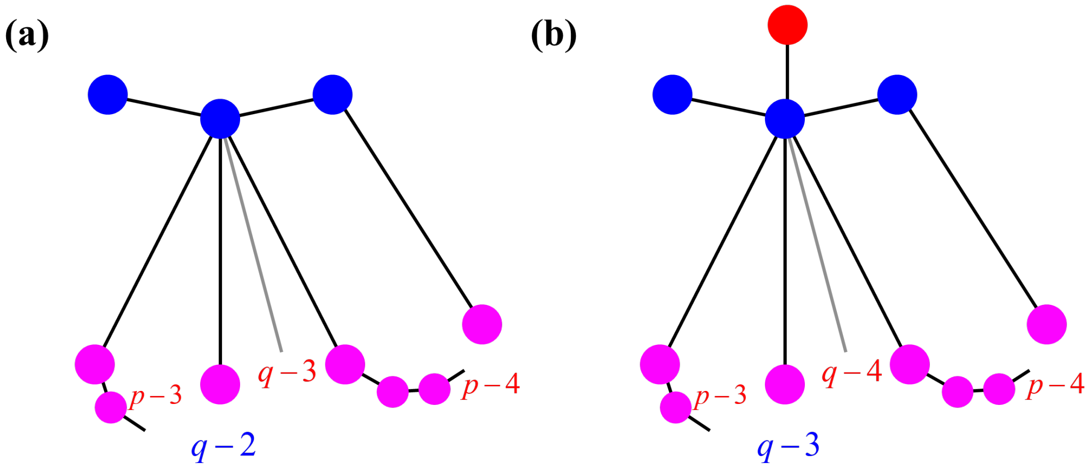

Fig. S3. The schematic diagram of the connection relationship of two types of lattice sites. (a). **a** lattice sites. (b). **b** lattice sites.

When the number of layers is more than 2, the above recursion (Eq. (S2)) can be further

simplified. In this case, we consider $b_i / a_i \approx R$ and have

$$\frac{a_{i+1}}{b_{i+1}} = \frac{a_i((p-3)(q-3)+p-4)+b_i((p-3)(q-4)+p-4)}{a_i(q-2)+b_i(q-3)};$$
$$R = \frac{R((p-3)(q-3)+p-4)+((p-3)(q-4)+p-4)}{R(q-2)+(q-3)}. \tag{S3}$$

And we can get

$$R = \frac{(p-4)+\sqrt{(p-4)^2+4\frac{((p-3)(q-4)+p-4)}{q-2}}}{2}, \tag{S4}$$

which represents the relationship between **a** lattice sites and **b** lattice sites as the number of layers increases.

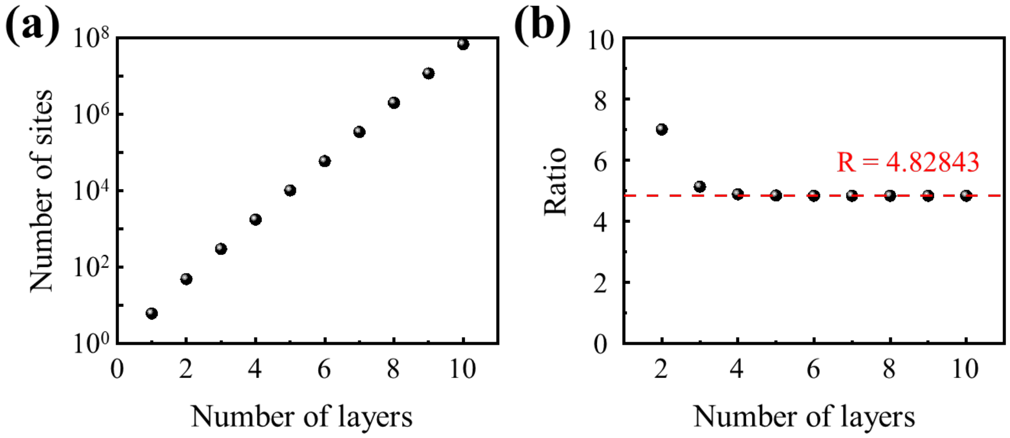

Fig. S4. The numerical relationship of the sites in the hyperbolic lattice. (a). The total number of sites of the hyperbolic lattices with different number of layers. (b). The ratio between the edge and the total sites.

For the {6, 4} lattices mentioned in the main text, we can obtain the total number of the lattice sites with different number of layers. The corresponding number is plotted in Fig. S4(a). We can see that the total number of lattice sites exponentially increases as the number of layers increases.

By subtracting the number of sites in the previous layer to the number of sites in this layer, we can get the number of edge sites. Fig. S4(a) plots the ratio between the numbers of edge and bulk sites. We can see that the ratio is a constant value (R=4.82843) if the number of layers is more than 3. The ratio indicates the number of edge sites is always much larger than the bulk sites. That is to say, more than 82.85% (4.83/5.83) lattice sites are the edge sites. So, the utilization rate of edge sites is greatly improved because of the geometric property of hyperbolic lattices.

S3. Design principle and structural parameters of the hyperbolic photonic structure.

S3.1. The derivation of the effective tight-binding Hamiltonian of coupled ring resonators.

It is well known that the momentum-space Hamiltonian of the lattice model with translational

symmetry in Euclidean space can be mapped to the transfer matrix of coupled optical ring resonators. In this case, the real space Hamiltonian can be easily obtained by performing Fourier transform on the *k*-space Hamiltonian [S2]. However, such a method is no longer hold in systems without Abelian translational symmetries. Therefore, it is necessary to provide a more general method to derive the effective tight-binding Hamiltonian of coupled ring resonators in hyperbolic lattices.

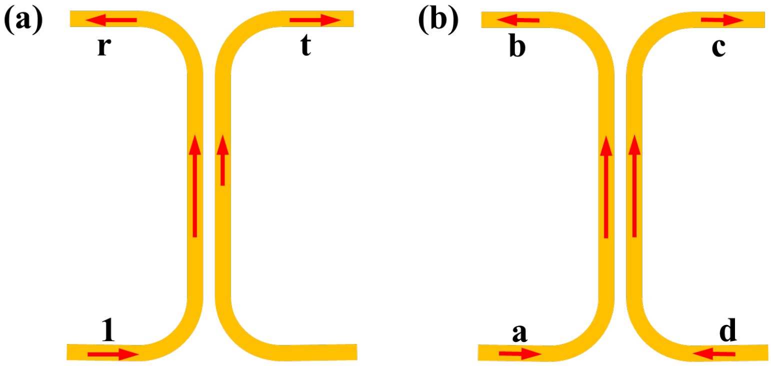

Fig. S5. The relationship between waveguide couplings. (a) Transmission situation when light is incident from the lower left corner. r is the reflection coefficient; t is the transmission coefficient. (b) Scattering matrix diagram when light passes through the waveguide coupler.

Firstly, we illustrate the coupling behavior between two nearby waveguides. When two waveguides are close to each other, as shown in Fig. S5(a), the electromagnetic wave can couple from one waveguide to the other. In this case, we assume that the amplitude of the injected wave is 1. After passing through the waveguide coupler, the wave amplitude in the injected waveguide is *r*; and the wave amplitude coupled to the other waveguide is *t*. Here, the backward scattering and losses in two waveguides are neglected, making the coupling between two spin modes (clockwise and counterclockwise eigenmodes of each onsite resonator) become zero. Therefore, according to the law of energy conservation, we have $|r|^2 + |t|^2 = 1$. For simplicity, we represent *r* and *t* with a single parameter θ as $r = cos\,\theta$, $t = -i\,sin\,\theta$. Duo to the reciprocity, the light entering the waveguide coupler from the other direction has the same transmittance and reflectance. Therefore, the light passing through the coupler has the following relationship

$$\begin{bmatrix} b \\ c \end{bmatrix} = \begin{bmatrix} r & t \\ t & r \end{bmatrix} \begin{bmatrix} a \\ d \end{bmatrix} = \begin{bmatrix} cos\,\theta & -i\,sin\,\theta \\ -i\,sin\,\theta & cos\,\theta \end{bmatrix} \begin{bmatrix} a \\ d \end{bmatrix}, \tag{S5}$$

where a, b, c and d correspond to wave amplitudes of four ports, as shown in Fig. S5(b). In this case, the scattering matrix of two coupled waveguides is represented by

$$\hat{S}(\theta) = \begin{bmatrix} cos\,\theta & -i\,sin\,\theta \\ -i\,sin\,\theta & cos\,\theta \end{bmatrix}. \tag{S6}$$

Using the above proposed scattering matrix, the scattering equation of two coupled onsite rings through a single linking ring (as shown in Fig. S6) in the counterclockwise-spin subspace can be described by

$$\begin{bmatrix} a_1' \\ s_1' \end{bmatrix} = \hat{S}(\theta) \begin{bmatrix} a_1 \\ s_1 \end{bmatrix}$$
$$\begin{bmatrix} a_2' \\ s_2' \end{bmatrix} = \hat{S}(\theta) \begin{bmatrix} a_2 \\ s_2 \end{bmatrix} \tag{S7}$$

where the amplitudes at different ports satisfy the relationships of $a_1' = a_1 e^{-i\xi}$, $s_1' =$

$s_2 e^{-i(\Phi+\phi)}$,$a_2^{'} = a_2 e^{-i\xi}$, and$s_2^{'} = s_1 e^{-i(\Phi-\phi)}$ with ξ and $\Phi \pm \varphi$ being the propagation phases as marked in Fig. S4. It is noted that the propagation phases and the parameter θ satisfy the following relationships with $\xi = 2\pi\delta v/FSR_a$, $\Phi = \pi + 2\pi\delta v/FSR_s$, and $\theta = \sqrt{4\pi J/FSR_a}$, where $FSR_a \sim 1/L_a$ and FSR_s represents the free spectral range of the onsite ring and the linking ring. δv represents the difference between the operating frequency and the resonant frequency of the onsite ring. Combining the Eq. S6 and Eq. S7, we can get

$$\begin{cases} a_2 \sin\xi = a_1 e^{-i\Phi}(\sin\Phi + \sin(\xi - \Phi)\cos\theta) \\ a_2 e^{i\Phi}(-\sin\Phi\cos\theta + \sin(\xi + \Phi)) = a_1 \sin\xi\cos\theta \end{cases} \tag{S8}$$

Here, we assume that the system stays in the weak coupling condition and possesses a small frequency shift, that is $4\pi J/FSR_a \sim 2\pi\delta v/FSR_a \sim \pi\delta v/FSR_s \sim \eta$ with η being a small parameter. Therefore, Eq. S8 can be simplified approximately to

$$\begin{cases} a_2 \delta v = a_1 e^{-i\Phi} J \\ a_2 e^{i\Phi} J = a_1 \delta v \end{cases}. \tag{S9}$$

Eq. S9 can be further written into a matrix form as:

$$\begin{bmatrix} 0 & Je^{i\phi} \\ Je^{-i\phi} & 0 \end{bmatrix}\begin{bmatrix} a_1 \\ a_2 \end{bmatrix} = \delta v \begin{bmatrix} a_1 \\ a_2 \end{bmatrix}, \tag{S10}$$

where the effective Hamiltonian is written as:

$$\hat{H} = \begin{bmatrix} 0 & Je^{i\phi} \\ Je^{-i\phi} & 0 \end{bmatrix} \tag{S11}$$

with δv being the effective eigenvalue. From above results, we can see that the coupling strength between two onsite rings is determined by the coupling angle of the scattering matrix. And, the coupling phase is determined by the propagation phase of ϕ.

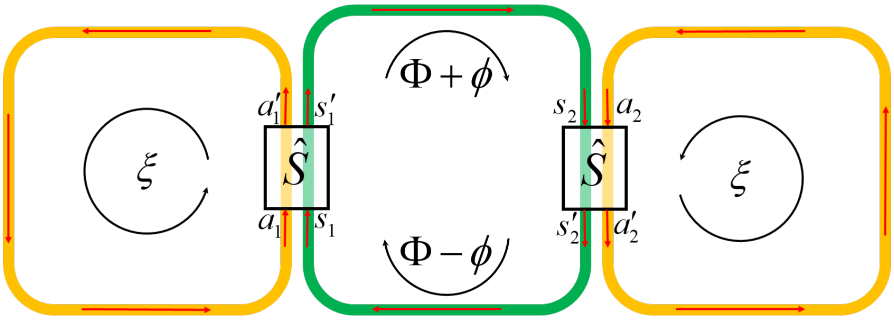

Fig. S6. The illustration of two coupled onsite rings through a linking ring. a_1, a_2 are used to describe the intensity of light in the site ring, s_1, s_2 is used to describe the intensity of light in the link ring. ξ represents the phase of light after one loop in the site ring, Φ represents the phase of light after half a loop in the linking ring, and ϕ represents the phase difference caused by the coupling region being offset from its symmetric position.

Next, we focus on the derivation of the effective Hamiltonian of six onsite resonators coupled by a single linking ring, as shown in Fig. S7. The scattering matrix of each onsite ring coupled to the linking ring is expressed as:

$$\begin{bmatrix} a_i^{'} \\ s_i^{'} \end{bmatrix} = \begin{bmatrix} \cos\theta & -i\sin\theta \\ -i\sin\theta & \cos\theta \end{bmatrix}\begin{bmatrix} a_i \\ s_i \end{bmatrix} \text{ with } i = 1,2,3,\cdots,6 \tag{S12}$$

with $a_i^{'} = a_i e^{-i\xi}$,$s_i^{'} = s_{i+1} e^{-i\Phi_i}$. We expand two scattering matrixes of the *i*-th and *i*+1-th rings as:

$$\begin{cases} is_i \sin\theta = a_i\left(\cos\theta - e^{-i\xi}\right) \\ is_{i+1}e^{-i\Phi_i}\sin\theta = a_i \sin^2\theta + is_i \sin\theta\cos\theta \\ is_{i+1}\sin\theta = a_{i+1}\left(\cos\theta - e^{-i\xi}\right) \\ is_{i+2}e^{-i\Phi_{i+1}}\sin\theta = a_{i+1}\sin^2\theta + is_{i+1}\sin\theta\cos\theta \end{cases} . \tag{S13}$$

It is clearly shown that, by eliminating the co-amplitudes of s_{i+1} and s_i, the amplitudes of a_i and a_i satisfy the following relationship of

$$a_{i+1}\left(\cos\theta - e^{-i\xi}\right)e^{-i\Phi_i} = a_i\left(1 - \cos\theta\, e^{-i\xi}\right) \tag{S14}$$

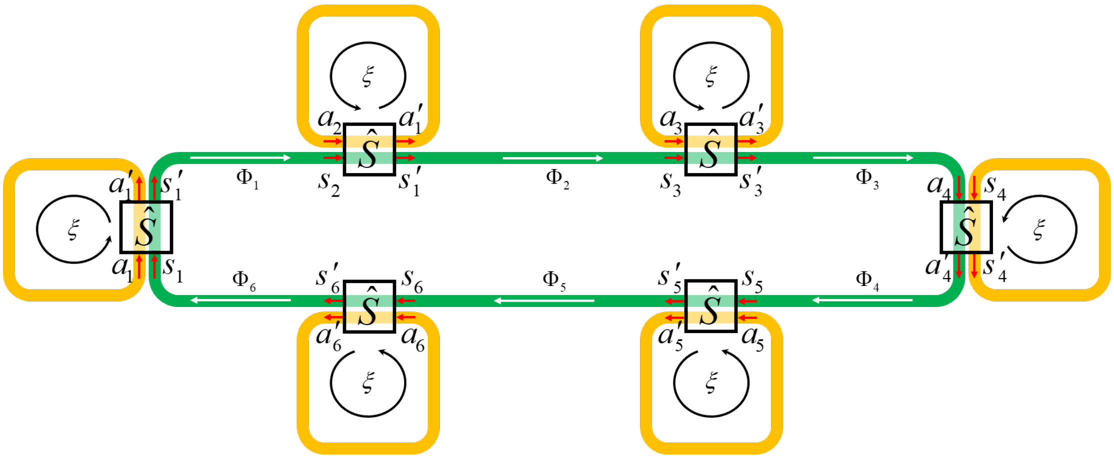

Fig. S7. The amplitude relationship of a link ring connected to N site rings. a_i represents the amplitude of the wave from the i-th input point entering the coupling region, a_i' represents the amplitude of the wave from the i-th output point exiting the coupling region. ξ represents the phase accumulated when the light circulates around the *i*-th site rings. s_i represents the amplitude of the wave entering the coupling region from the linking ring to the i-th input point. s_i' represents the amplitude of the wave exiting the coupling region from the *i*-th output point to the linking ring. Φ_i represents the phase accumulated by the wave after leaving the coupling region of the i-th output point and before entering the coupling region of the (*i*+1)-th input point in the linking ring.

Eq. S14 can be re-expressed as:

$$a_i\left(i\sin\frac{\xi}{2} + \sin^2\frac{\theta}{2}e^{-i\xi/2}\right) = a_{i+1}\left(i\sin\frac{\xi}{2} + \sin^2\frac{\theta}{2}e^{-i\xi/2}\right)e^{-i\Phi_i} - 2a_{i+1}\sin^2\frac{\theta}{2}\cos\frac{\xi}{2}e^{-i\Phi_i} \tag{S15}$$

Using Eq. S15, we can obtain following six equations (with *i*=1, 2, 3, 4, 5, 6) as:

$$\begin{cases} a_1\left(i\sin\frac{\xi}{2} + \sin^2\frac{\theta}{2}e^{-i\xi/2}\right) = a_2\left(i\sin\frac{\xi}{2} + \sin^2\frac{\theta}{2}e^{-i\xi/2}\right)e^{-i\Phi_1} - 2a_2\sin^2\frac{\theta}{2}\cos\frac{\xi}{2}e^{-i\Phi_1} \\ a_2\left(i\sin\frac{\xi}{2} + \sin^2\frac{\theta}{2}e^{-i\xi/2}\right) = a_3\left(i\sin\frac{\xi}{2} + \sin^2\frac{\theta}{2}e^{-i\xi/2}\right)e^{-i\Phi_2} - 2a_3\sin^2\frac{\theta}{2}\cos\frac{\xi}{2}e^{-i\Phi_2} \\ a_3\left(i\sin\frac{\xi}{2} + \sin^2\frac{\theta}{2}e^{-i\xi/2}\right) = a_4\left(i\sin\frac{\xi}{2} + \sin^2\frac{\theta}{2}e^{-i\xi/2}\right)e^{-i\Phi_3} - 2a_4\sin^2\frac{\theta}{2}\cos\frac{\xi}{2}e^{-i\Phi_3} \\ a_4\left(i\sin\frac{\xi}{2} + \sin^2\frac{\theta}{2}e^{-i\xi/2}\right) = a_5\left(i\sin\frac{\xi}{2} + \sin^2\frac{\theta}{2}e^{-i\xi/2}\right)e^{-i\Phi_4} - 2a_5\sin^2\frac{\theta}{2}\cos\frac{\xi}{2}e^{-i\Phi_4} \\ a_5\left(i\sin\frac{\xi}{2} + \sin^2\frac{\theta}{2}e^{-i\xi/2}\right) = a_6\left(i\sin\frac{\xi}{2} + \sin^2\frac{\theta}{2}e^{-i\xi/2}\right)e^{-i\Phi_6} - 2a_5\sin^2\frac{\theta}{2}\cos\frac{\xi}{2}e^{-i\Phi_5} \\ a_6\left(i\sin\frac{\xi}{2} + \sin^2\frac{\theta}{2}e^{-i\xi/2}\right) = a_1\left(i\sin\frac{\xi}{2} + \sin^2\frac{\theta}{2}e^{-i\xi/2}\right)e^{-i\Phi_1} - 2a_1\sin^2\frac{\theta}{2}\cos\frac{\xi}{2}e^{-i\Phi_6} \end{cases} \tag{S16}$$

Combing these six equations, we have

$$a_1\left(i\sin\frac{\xi}{2} + \sin^2\frac{\theta}{2}e^{-i\xi/2}\right)$$
$$= a_1\left(i\sin\frac{\xi}{2} + \sin^2\frac{\theta}{2}e^{-i\xi/2}\right)e^{-i\sum_{j=1}^{6}\Phi_j} - 2a_1\sin^2\frac{\theta}{2}\cos\frac{\xi}{2}e^{-i\sum_{j=1}^{6}\Phi_j}$$

$$-2a_6\, sin^2\frac{\theta}{2}cos\frac{\xi}{2}e^{-i\sum_{j=1}^{5}\Phi_j} - \cdots - 2a_3\, sin^2\frac{\theta}{2}cos\frac{\xi}{2}e^{-i(\Phi_1+\Phi_2)} - 2a_2\, sin^2\frac{\theta}{2}cos\frac{\xi}{2}e^{-i\Phi_1} \quad \text{(S17)}$$

For the sake of simplicity, we make the following substitutions with $\sum_{j=1}^{m-1}\Phi_j = \Phi/2 - \varphi_{1m}$, $\sum_{j=m}^{N}\Phi_j = \Phi/2 - \varphi_{m1} = \Phi/2 + \varphi_{1m}$, where $\Phi = \sum_{j=1}^{N}\Phi_j$ is the phase for the light goes through the entire ring. Then, the above equation can be simplified as:

$$a_1\left(i\, sin\frac{\xi}{2} + sin^2\frac{\theta}{2}e^{-i\xi/2}\right) = a_1\left(i\, sin\frac{\xi}{2} + sin^2\frac{\theta}{2}e^{-i\xi/2}\right)e^{-i\Phi} - 2a_1\, sin^2\frac{\theta}{2}cos\frac{\xi}{2}e^{-i\Phi}$$

$$-2a_6\, sin^2\frac{\theta}{2}cos\frac{\xi}{2}e^{-i\left(\frac{\Phi}{2}-\varphi_{1,N}\right)} - \cdots - 2a_3\, sin^2\frac{\theta}{2}cos\frac{\xi}{2}e^{-i\left(\frac{\Phi}{2}-\varphi_{1,3}\right)} - 2a_2\, sin^2\frac{\theta}{2}cos\frac{\xi}{2}e^{-i(\Phi/2-\varphi_{1,2})}$$

(S18)

Similar to the above case with two coupled onsite rings, Eq. S18 can be expressed as:

$$\delta v a_1 = Ja_6 e^{i\varphi_{1,6}} + \cdots + Ja_3 e^{i\varphi_{1,3}} + Ja_2 e^{i\varphi_{1,2}}, \quad \text{(S19)}$$

when the system is in the weak coupling condition and possesses a small frequency shift.

Similar to the case of a_1, the amplitudes at other onsite rings can also be expressed as

$$\delta v a_i = Ja_6 e^{i\varphi_{i,6}} + \cdots + Ja_{i+1}e^{i\varphi_{i,i+1}} + Ja_{i-1}e^{i\varphi_{i,i-1}} + \cdots + Ja_2 e^{i\varphi_{i,2}} + Ja_1 e^{i\varphi_{i,1}} \quad \text{(S20)}$$

Writing Eq. S20 into a matrix form, we have

$$\begin{bmatrix} 0 & Je^{i\varphi_{1,2}} & Je^{i\varphi_{1,3}} & Je^{i\varphi_{1,4}} & Je^{i\varphi_{1,5}} & Je^{i\varphi_{1,6}} \\ Je^{i\varphi_{2,1}} & 0 & Je^{i\varphi_{2,3}} & Je^{i\varphi_{2,4}} & Je^{i\varphi_{2,5}} & Je^{i\varphi_{2,6}} \\ Je^{i\varphi_{3,1}} & Je^{i\varphi_{3,2}} & 0 & Je^{i\varphi_{3,4}} & Je^{i\varphi_{3,5}} & Je^{i\varphi_{3,6}} \\ Je^{i\varphi_{4,1}} & Je^{i\varphi_{4,2}} & Je^{i\varphi_{4,3}} & 0 & Je^{i\varphi_{4,5}} & Je^{i\varphi_{4,6}} \\ Je^{i\varphi_{5,1}} & Je^{i\varphi_{5,2}} & Je^{i\varphi_{5,3}} & Je^{i\varphi_{5,4}} & 0 & Je^{i\varphi_{5,6}} \\ Je^{i\varphi_{6,1}} & Je^{i\varphi_{6,2}} & Je^{i\varphi_{6,3}} & Je^{i\varphi_{6,4}} & Je^{i\varphi_{6,5}} & 0 \end{bmatrix} \begin{bmatrix} a_1 \\ a_2 \\ a_3 \\ a_4 \\ a_5 \\ a_6 \end{bmatrix} = \delta v \begin{bmatrix} a_1 \\ a_2 \\ a_3 \\ a_4 \\ a_5 \\ a_6 \end{bmatrix} \quad \text{(S21)}$$

By considering the case with $\varphi_{i,j} = -\varphi_{j,i}$, the above matrix can be written as:

$$\begin{bmatrix} 0 & Je^{i\varphi_{1,2}} & Je^{i\varphi_{1,3}} & Je^{i\varphi_{1,4}} & Je^{i\varphi_{1,5}} & Je^{i\varphi_{1,6}} \\ Je^{-i\varphi_{1,2}} & 0 & Je^{i\varphi_{2,3}} & Je^{i\varphi_{2,4}} & Je^{i\varphi_{2,5}} & Je^{i\varphi_{2,6}} \\ Je^{-i\varphi_{1,3}} & Je^{-i\varphi_{2,3}} & 0 & Je^{i\varphi_{3,4}} & Je^{i\varphi_{3,5}} & Je^{i\varphi_{3,6}} \\ Je^{-i\varphi_{1,4}} & Je^{-i\varphi_{2,4}} & Je^{-i\varphi_{3,4}} & 0 & Je^{i\varphi_{4,5}} & Je^{i\varphi_{4,6}} \\ Je^{-i\varphi_{1,5}} & Je^{-i\varphi_{2,5}} & Je^{-i\varphi_{3,5}} & Je^{-i\varphi_{4,5}} & 0 & Je^{i\varphi_{5,6}} \\ Je^{-i\varphi_{1,6}} & Je^{-i\varphi_{2,6}} & Je^{-i\varphi_{3,6}} & Je^{-i\varphi_{4,6}} & Je^{-i\varphi_{5,6}} & 0 \end{bmatrix} \begin{bmatrix} a_1 \\ a_2 \\ a_3 \\ a_4 \\ a_5 \\ a_6 \end{bmatrix} = \delta v \begin{bmatrix} a_1 \\ a_2 \\ a_3 \\ a_4 \\ a_5 \\ a_6 \end{bmatrix}, \quad \text{(S22)}$$

which is the effective Hamiltonian of six coupled onsite rings. Therefore, based on the scenario described in Eq. S22, we know that the S-matrix can be tuned by manipulating the separation distance between the site rings and the link rings, thereby controlling the coupling strength J. A smaller separation distance results in a stronger coupling strength J, while a larger separation distance leads to a weaker coupling strength J. Additionally, we can tune the coupling phase $\varphi_{i,j}$ between different site rings by manipulating the propagation phase Φ_i of the light wave within the link rings. We can design spatial positions of six onsite rings so that the phase difference between each adjacent coupling ring is a constant of $\Phi_i = \pi/6, i = 1,2,3,4,5,6$. Therefore, the Hamiltonian equation becomes

$$\begin{bmatrix} 0 & Je^{i\pi/3} & Je^{i\pi/6} & J & Je^{-i\pi/6} & Je^{-i\pi/3} \\ Je^{-i\pi/3} & 0 & Je^{i\pi/3} & Je^{i\pi/6} & J & Je^{-i\pi/6} \\ Je^{-i\pi/6} & Je^{-i\pi/3} & 0 & Je^{i\pi/3} & Je^{i\pi/6} & J \\ J & Je^{-i\pi/6} & Je^{-i\pi/3} & 0 & Je^{i\pi/3} & Je^{i\pi/6} \\ Je^{i\pi/6} & J & Je^{-i\pi/6} & Je^{-i\pi/3} & 0 & Je^{i\pi/3} \\ Je^{i\pi/3} & Je^{i\pi/6} & J & Je^{-i\pi/6} & Je^{-i\pi/3} & 0 \end{bmatrix} \begin{bmatrix} a_1 \\ a_2 \\ a_3 \\ a_4 \\ a_5 \\ a_6 \end{bmatrix} = \delta v \begin{bmatrix} a_1 \\ a_2 \\ a_3 \\ a_4 \\ a_5 \\ a_6 \end{bmatrix} \quad \text{(S23)}$$

Based on the relationship of $\delta v = \omega - \omega_0$, Eq. S23 can be expressed as:

$$\begin{bmatrix} \omega_0 & Je^{i\pi/3} & Je^{i\pi/6} & J & Je^{-i\pi/6} & Je^{-i\pi/3} \\ Je^{-i\pi/3} & \omega_0 & Je^{i\pi/3} & Je^{i\pi/6} & J & Je^{-i\pi/6} \\ Je^{-i\pi/6} & Je^{-i\pi/3} & \omega_0 & Je^{i\pi/3} & Je^{i\pi/6} & J \\ J & Je^{-i\pi/6} & Je^{-i\pi/3} & \omega_0 & Je^{i\pi/3} & Je^{i\pi/6} \\ Je^{i\pi/6} & J & Je^{-i\pi/6} & Je^{-i\pi/3} & \omega_0 & Je^{i\pi/3} \\ Je^{i\pi/3} & Je^{i\pi/6} & J & Je^{-i\pi/6} & Je^{-i\pi/3} & \omega_0 \end{bmatrix} \begin{bmatrix} a_1 \\ a_2 \\ a_3 \\ a_4 \\ a_5 \\ a_6 \end{bmatrix} = \omega \begin{bmatrix} a_1 \\ a_2 \\ a_3 \\ a_4 \\ a_5 \\ a_6 \end{bmatrix} \quad \text{(S24)}$$

It is clearly shown that the coupling term can be divided into three cases. These three cases correspond to the *NN* hopping of $Je^{\pm i\pi/3}$, the *NNN* hopping of $Je^{\pm i\pi/3}$, and the *NNNN* hopping of J, respectively. Therefore, the Hamiltonian in the tight-binding model can be expressed as:

$$\hat{H} = \sum_{i=1}^{6} \omega_0 a_i^\dagger a_i + J\,exp(i\tfrac{\pi}{3}) a_i^\dagger a_{i+1} + J\,exp(i\tfrac{\pi}{6}) a_i^\dagger a_{i+2} + J a_i^\dagger a_{i+3} + h.c. \quad \text{(S25)}$$

In the above derivation, we only consider the system in the counterclockwise-spin subspace. Similarly, the effective Hamiltonian in the clockwise-spin subspace is described by

$$\hat{H} = \sum_{i=1}^{6} \omega_0 a_i^\dagger a_i + J\,exp(-i\tfrac{\pi}{3}) a_i^\dagger a_{i+1} + J\,exp(-i\tfrac{\pi}{6}) a_i^\dagger a_{i+2} + J a_i^\dagger a_{i+3} + h.c. \quad \text{(S26)}$$

S3.2. The structural parameters of our designed hyperbolic topological insulator.

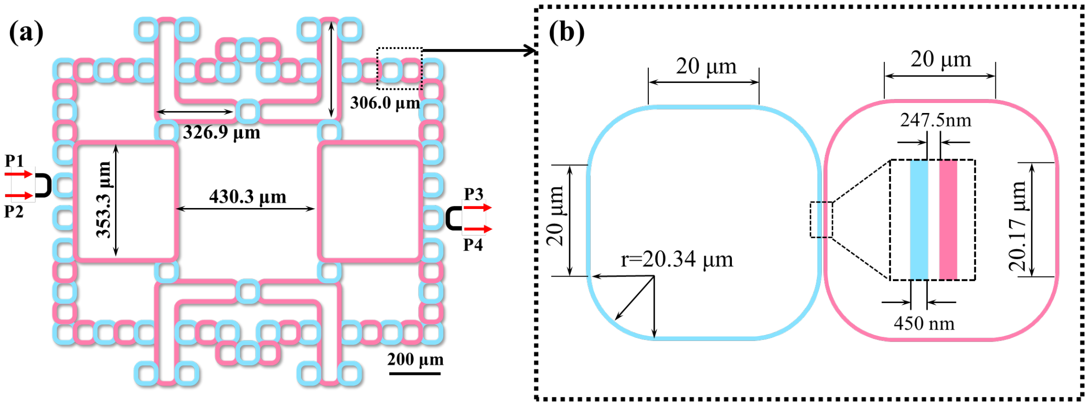

Fig. S8. The schematic diagram of the hyperbolic photonic topological insulator and its detailed parameters. (a). The schematic diagram of the designed hyperbolic photonic topological insulator with two layers. (b). The illustration of detailed geometric parameters, including radius and widths of onsite and small-linking rings and the distance between linking rings and onsite rings.

In this section, we construct the {6,4} hyperbolic topological lattice by evanescently coupled optical resonators, where the schematic diagram of designed optical structure with two layers is shown in Fig. S8(a). In this structure, optical ring resonators can be divided into the onsite resonators (the blue blocks) and linking rings (the pink blocks) according to their functionalities. Specifically, different onsite resonators have exactly the same geometric parameters, ensuring the same resonant frequency and free spectral range (FSR) of all onsite resonators. The suitably designed linking rings are used to couple different onsite resonators to implement required site couplings. Due to the aperiodicity of hyperbolic lattices, the size and coupling pattern of linking rings should be suitably designed. Here, the small-size linking ring is used to couple two boundary-onsite resonators to

simulate *NN* hoppings. In addition, six onsite resonators are coupled by a single large-size linking ring to realize required *NN*, *NNN*, and *NNNN* couplings. Geometric parameters of large coupling rings are illustrated in Fig. S8(a). Fig. S8(b) presents detailed parameters of radius and widths for site rings and small-size linking rings, as well as the distance between linking rings and onsite rings. Through the appropriate setting of spatial positions and coupling patterns of onsite resonators and linking rings, the eigenequation of our designed evanescently coupled ring-resonator array is identical with that of the topological hyperbolic lattice model.

S3.3. The interferences of the big link rings in the total transmission spectra.

The transmission spectra in these FSRs are different from each other. The main reason is that there are some link rings with larger perimeters used in the hyperbolic structure, as highlighted in the yellow lines of Fig. S9.

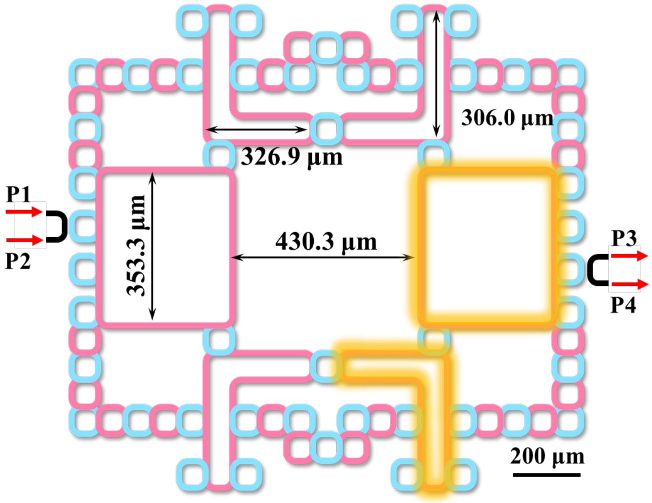

Fig. S9. The hyperbolic photonic structure. Two kinds of big link rings are highlighted in yellow lines.

In fact, we would like to point out that the ideal transmission spectra of the hyperbolic photonic structure are same to the transmission spectra of a single site ring, as plotted in Fig. S10(a). Due to the usage of the big link rings to satisfy the phase relation of the hyperbolic TI, some interferences have to be introduced into the total transmission spectra. As shown in Figs. S10(b) and S10(c), the two kinds of link rings bring the two kinds of modulation for the transmission with different periods. Thus, the total transmission of the hyperbolic structure can be obtained by multiplying these transmissions in Figs. S10(a)-S10(c). The total transmission is plotted in Fig. S10(d). We can see that the lattice spectra in the different FSRs are not same to each other.

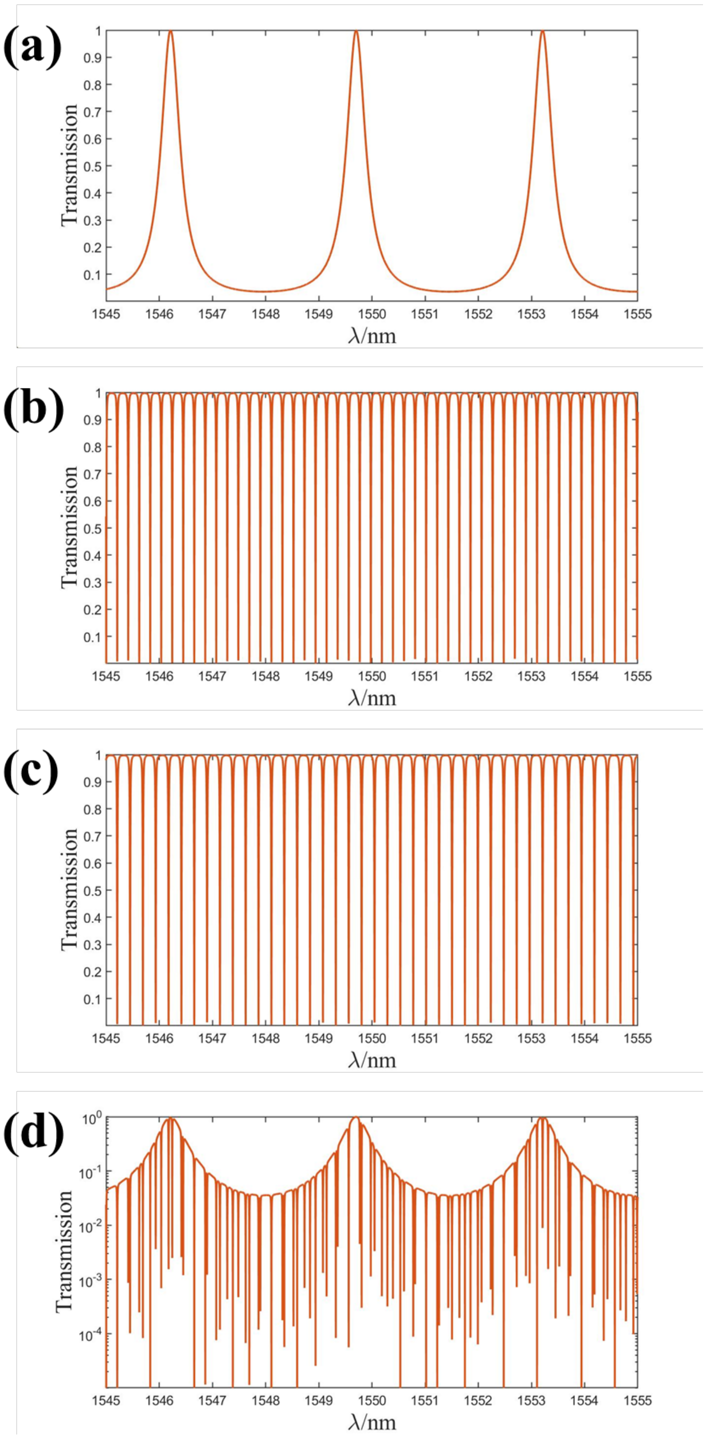

Fig. S10. The theoretical explanation of the difference between these lattice spectra in FSRs. (a). The transmission spectra of a single site ring. (b). The transmission spectra of the first kind of link ring. (c). The transmission spectra of the second kind of link ring. (b). The total transmission spectra of the hyperbolic structure.

S4. Numerical simulations of the topological hyperbolic photonic insulator.

To further demonstrate topological effects of one-way propagation, we perform the full-wave simulation of wave propagation in the hyperbolic photonic insulator using finite element methods. In the simulation, we can effectively predict the performance of real 3D optical structures by setting

the appropriate optical parameters in the two-dimensional (2D) simulation. Here, the effective refractive index of the optical waveguide (environment) is set as 2.832 (1.2) to simulate the 3D silicon waveguides embedding into the background of silica.

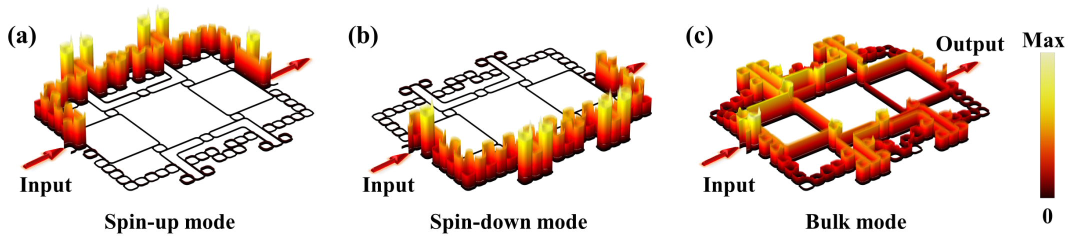

Fig. S11. Simulation results of steady-state distributions of electric fields. Simulation results by exciting (a) the topological spin-up mode and (b) the topological spin-down mode with the wavelength being 1549.7 nm. (c). The steady-state distribution of electric fields by exciting the topological bulk mode with the wavelength being 1550.3 nm.

Figs. S11(a) and S11(b) present the steady-state distributions of electric fields by exciting the topological spin-up and spin-down modes at 1549.7 nm (equaling to the central wavelength of $2\pi c_0/\omega_0$). It is shown that the one-way transport of input signals along the edge with pseudospin-dependent propagation directions appears, showing key behaviors of topological edge states. For comparison, we also calculate the steady-state distribution of electric fields by exciting the bulk state at 1550.3 nm, as shown in Fig. S11(c). It is shown that the input electric fields can permeate into the bulk, and the bidirectional edge propagation also appears, meaning the excitation of trivial bulk and edge states. These simulation results demonstrate the correctness on the implementation of hyperbolic topological insulators by coupled optical-ring resonators.

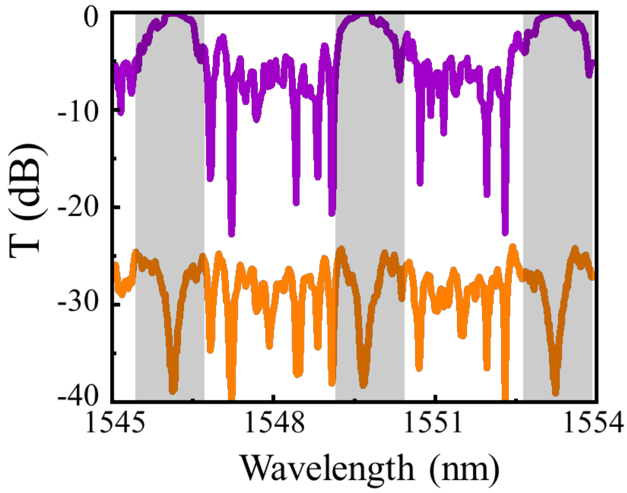

Fig. S12. Simulated transmission spectra by exciting the topological spin-up mode. Purple and orange lines correspond to results of output ports at clockwise and counterclockwise positions with respect to the input port, respectively.

Furthermore, Fig. S12 displays the simulated transmission spectrum by exciting the spin-up mode. Purple and orange lines correspond to results from output ports at clockwise and

counterclockwise positions, respectively. Around the central frequency of each FSR (highlight by dark regions), there is a large transmission platform from the clockwise (counterclockwise) output port under the excitation of the spin-up mode, showing the existence of a pseudospin-dependent topological edge state. Away from the central frequency, a large number of transmission peaks appear and transmissions of the clockwise (counterclockwise) output port under the excitation of the spin-up mode are significantly decreased, indicating the excitation trivial bulk eigenmodes. Additionally, due to the mirror symmetry of the structure, the corresponding results of the spin-down mode is expected to be similar to that of spin-up mode.

Here, we would like to discuss the theoretical model to consider the brightness of the hyperbolic and Euclidean quantum source. First, we present the generation efficiency of signal-idler photon pairs in a single-ring resonator [Opt. Express 22, 2620-2631 (2014)]:

$$\mathrm{RG} = \frac{2\hbar \mathrm{P}_{\mathrm{in}}^2 \gamma^2 \mathrm{v}_{\mathrm{g}}^4 \mathrm{Q}^7}{\mathrm{L}^2 \omega_{\mathrm{p}}^2 \mathrm{Q}_{\mathrm{e}}^4}, \tag{S27}$$

where P_{in} is pump power, γ is the nonlinear coupling constant of the waveguide, v_g is the group velocity of the light propagating in the cavity, L is the cavity circumference, ω_p is the angular frequency of the pump light, Q is the Q factor of the cavity mode, Q_e is the external Q factor of the cavity mode. From Eq. S32, we know that under the condition of consistent parameters such as the shape, circumference, and Q factor of the ring resonator, the generation rate RG is proportional to the pump power.

Hence, if each ring in Euclidean and hyperbolic lattices possesses the same capacity to generate photon pairs and the same loss, the brightness of the quantum source constructed by Euclidean and hyperbolic lattices can be provided in following derivation.

Next, we theoretically derive the photon generation rate of multi-ring quantum sources [arXiv.2311.17313] [arXiv.2404.17570]. Both Euclidean and hyperbolic quantum sources consist of alternating resonant and non-resonant rings. We assume that the non-resonant rings only transmit the pump laser and generated signal-idler photons, without participating in the nonlinear process of spontaneous four-wave mixing. Consequently, the models of hyperbolic and Euclidean quantum sources can be simplified to a system of *N* rings and a bus waveguide. And then, let's consider the case of generating a quantum source using multiple rings. We assume that the loss in each ring is δ. The incident pump power is P_{in}. Therefore, the power at the *i*-th ring is $P_i=P_{in}e^{(-\delta \cdot i)}$. Consequently, the generation rate RG of the *i*-th ring is

$$\mathrm{RG}_{\mathrm{i}} \propto \mathrm{P}_{\mathrm{in}}^2 \mathrm{e}^{-2\delta \mathrm{i}}. \tag{S28}$$

The photons generated at the *i*-th ring must pass through (*N*-*i*) additional rings to reach the output port. During this process, an additional loss occurs, which is quantified by $e^{-\delta(N-i)}$. Therefore, the photon generation at the output port due to the *i*-th ring is

$$\mathrm{RG}_{\mathrm{i}} \propto \mathrm{P}_{\mathrm{in}}^2 \mathrm{e}^{-\delta(\mathrm{N}+\mathrm{i})}. \tag{S29}$$

Therefore, the total generation efficiency from all rings is:

$$
\begin{aligned}
RG_{total} &\propto \sum_{i=0}^{N} P_{in}^{2} e^{-\delta(N+i)} \\
&= P_{in}^{2} e^{-\delta N} \sum_{i=0}^{N} e^{-\delta i} \\
&\approx P_{in}^{2} (e^{-\delta N} - e^{-2\delta N}) / \delta.
\end{aligned} \tag{S30}
$$

We would like to point out the number of rings (N) in Eqs. S34 and S35 presents the number of the edge rings of the hyperbolic and Euclidean quantum sources. Furthermore, according to hyperbolic (Euclidean) structure, the relationship between the numbers of total and edge rings of these two kinds of lattices are also expressed as:

$$
N_{hyperbolic} = 0.829 N_{total}, N_{Euclidean} = 4\sqrt{N_{total}} - 4. \tag{S31}
$$

Here, $N_{hyperbolic}$ is the number of the edge rings for hyperbolic lattices, $N_{Euclidean}$ is the number of the edge rings for Euclidean lattices, and N_{total} is the total number of the site rings for these lattices. We can see that $N_{hyperbolic}$ is in direct proportion to N_{total} (See detailed discussion in the S2 of Supplementary Materials). As for the Euclidean lattices, $N_{Euclidean}$ is in direct proportion to $\sqrt{N_{total}} - 1$.

To assess the efficiency of the quantum source in utilizing the rings, we can rewrite Eq. S36 in the following forms:

$$
\begin{aligned}
RG_{hyperbolic} &\propto P_{in}^{2} (e^{-0.829\delta N_{total}} - e^{-2\times 0.829\delta N_{total}}) / \delta; \\
RG_{Euclidean} &\propto P_{in}^{2} (e^{-\delta(4\sqrt{N_{total}}-4)} - e^{-2\delta(4\sqrt{N_{total}}-4)}) / \delta.
\end{aligned} \tag{S32}
$$

For comparing the brightness of Euclidean and hyperbolic quantum sources, we plot Eq. S32 in Fig. 1d in the main text. We can see that both Euclidean and hyperbolic quantum sources achieve the *same maximum* brightness by utilizing a *different* total number of rings. Herein, **the advantage of the hyperbolic quantum source is that it can attain optimal brightness with fewer rings**. Hyperbolic topological quantum source exhibits a significant reduction in using the total number of rings, compared to its Euclidean counterparts.

S5. The discussion of momentum matching of the hyperbolic and Euclidean lattices.

The conservation of momentum is essential in the SFWM process. For satisfying momentum conservation conditions, the phase matching is necessary, which can be expressed as:

$$
(k_s + k_i) - 2k_p = 2\gamma P. \tag{S33}
$$

where k_s, k_i, and k_p are the wave vectors of the signal, idler, and pump photons, respectively. γ is the nonlinear optical coefficient. P is the pump power.

Correspondingly, the group velocities of the signal and idler photons (v_{g1} and v_{g2}) can be expressed as:

$$
v_{g1} = \frac{\partial \omega_1}{\partial k_1} \approx \frac{-\Delta\omega}{k_s - k_p}, \tag{S34}
$$

$$v_{g2} = \frac{\partial \omega_2}{\partial k_2} \approx \frac{\Delta\omega}{k_i - k_p}, \tag{S35}$$

where ω_s, ω_i, and ω_p are the angular frequency of the signal, idler, and pump photons, respectively.

By substituting Eqs. S34 and S35 into Eq. S33, we can obtain:

$$\frac{1}{v_{g2}} - \frac{1}{v_{g1}} = \frac{2\gamma P}{\Delta\omega}. \tag{S36}$$

We can see that the momentum/phase matching condition is satisfied if the dispersion relationship of the edge states for the signal and idler photons satisfies Eq. S36.

Additionally, we have the nonlinear coefficient γ, which is obtained through numerical simulations with a value of approximately 97 $W^{-1}m^{-1}$. The pump power is about 10 mW. $\Delta\omega$ is $2\pi\times10^{11}$ Hz. The value on the right-hand side of Eq. S36 is approximately 3.09×10^{-12} $Hz^{-1}m^{-1}$. We find that the value of vg is about 0.5×10^{8} Hz·m. This means that the right-hand side of the equation is about four orders of magnitude smaller than the left-hand side, i.e., $\frac{1}{v_{g2}} \approx \frac{1}{v_{g1}} \gg \frac{2\gamma P}{\Delta\omega}$. Therefore, approximately speaking, only $\frac{1}{v_{g2}} = \frac{1}{v_{g1}}$ is required to achieve momentum matching. Thus, the phase matching condition can be approximated as:

$$(k_s + k_i) - 2k_p \approx 0. \tag{S37}$$

To compare the momentum matching effects between Euclidean and hyperbolic lattices, we calculate the eigenstates of the Euclidean lattice with ~300 lattice sites (Fig. S13a), the Euclidean lattice with ~4000 lattice sites (Fig. S13b), and the hyperbolic lattice with ~300 lattice sites (Fig. S13c). It can be seen that when the eigenvalue ε is in the frequency range from -1J to +1J, the wave functions of Euclidean and hyperbolic lattices are located at the edge. Their bandgap sizes are roughly equal, both being approximately 2J.

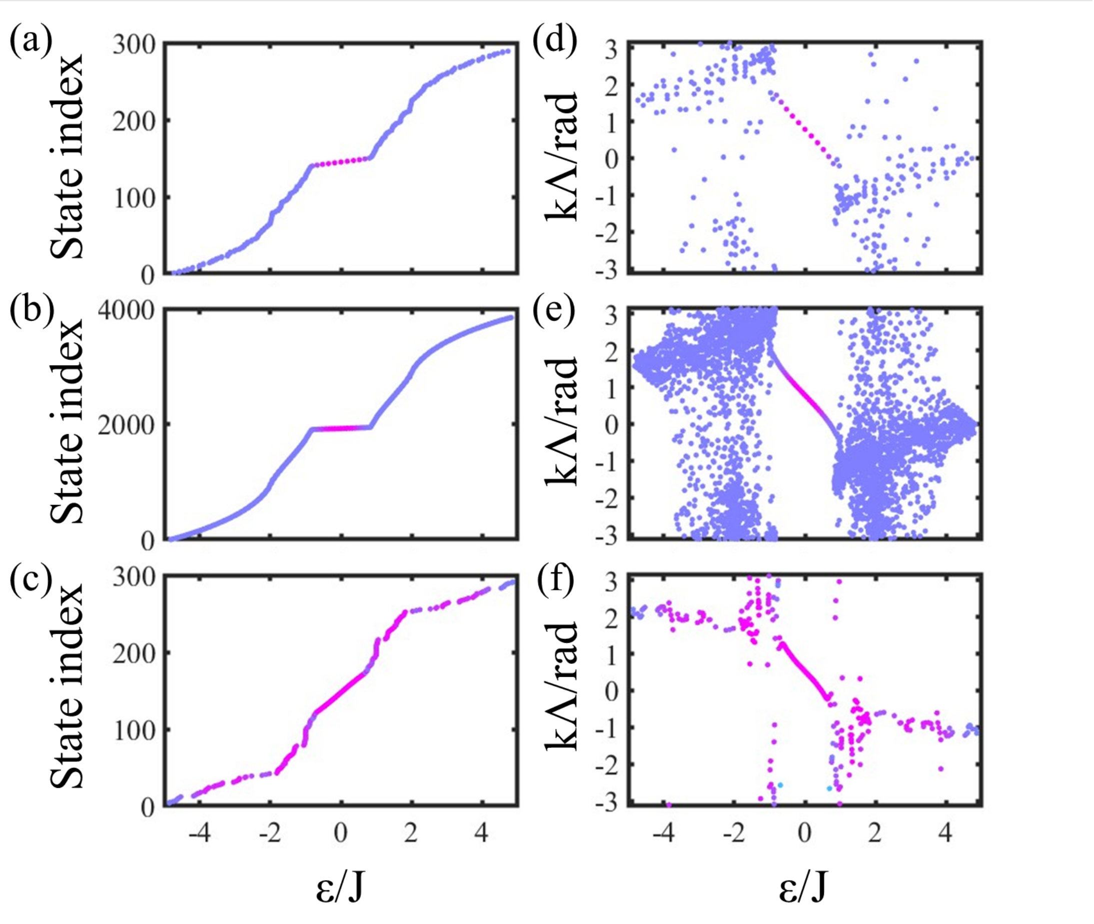

Fig. S13. The phase matching condition of the Euclidean and hyperbolic quantum sources. The eigenspectra of the Euclidean lattice with ~300 lattice sites (a), the Euclidean lattice with ~4000 lattice sites (b), and the hyperbolic lattice with ~300 lattice sites (c). (d-e). The corresponding simulated dispersion curve showing linear dispersion for the edge modes. Here k is the photon momentum and Λ is the lattice constant. kΛ is the phase difference between two neighboring site rings on the edge. The color of the scatter points represents localization strength of eigenstates on lattice sites at the edge, with pink indicating that the eigenstate is located at the boundary, and blue indicating that the eigenstate is within the bulk.

We also calculate the dispersion curves of Euclidean and hyperbolic lattices by extracting the phase difference between two neighboring sites on the edge. The simulated results are plotted in Figs. S13(d-e). It can be observed that for the Euclidean lattice, with a total of 300 lattice sites, the dispersion curve appears as a series of isolated points due to the small number of boundary sites (approximately 60). The gaps between these points can lead to a distortion of the linear dispersion, which may not satisfy the momentum matching conditions required for the quantum source (Eq. S37). Increasing the total number of lattice sites in the Euclidean lattice to approximately 4000 results in a denser set of edge state modes (approximately 250), leading to a better linear dispersion curve. On the other hand, for the hyperbolic lattice, a much smaller total number of lattice sites (approximately 300) can achieve a similar effect to that of a larger Euclidean lattice (with an edge mode count of approximately 250). Therefore, such a more efficient phase-matching curve can satisfy the SFWM process when the pump, as well as the signal and idler frequencies, correspond

to edge modes.

S6. The discussion of the brightness and CAR in different frequency channel.

We would like to thank the reviewer for bringing us your comment. Firstly, we would like to clarify that the free spectral range (FSR) of our structure is about 330 GHz. Secondly, by employing theoretical analysis and experimental verification, we find that there is no difference of the brightness in different frequency channels. However, we also find that when the frequencies of the signal and the idle photons are selected to be farther away from the pump photon frequency, the CAR of the quantum source increases. In the following, we would like to discuss these additional theoretical and experimental results.

1. In theory, we derive the frequency range of SFWM quantum sources generated by the Silicon waveguides. Similar to the SFWM process in the nonlinear fiber [S3], we calculate the spectral density of photon flux generated in the Silicon waveguide. In this work, the Silicon waveguide cross-section is 450 nm×220 nm. The TE mode of the Silicon waveguide is shown in Fig. S14(a). Furthermore, by simulation calculation, we can obtain the group velocity dispersion parameter $D = \frac{d\beta_1}{d\lambda} = \frac{2\pi c}{\lambda^2}\beta_2$, where λ is the wavelength, β_1 (β_2) is the first and second derivatives of the group velocity β with respect to the circular frequency ω, i.e. $\beta_1 = \frac{d\beta}{d\omega}$ ($\beta_2 = \frac{d^2\beta}{d\omega^2}$).

Consider a single frequency pump light incident into a silicon waveguide, in the SFWM process, the initial state is a vacuum state, denoted as $|0\rangle$. Using perturbation theory of quantum mechanics, the final output state after a silicon waveguide with length L is $|\psi(L)\rangle = |0\rangle + \int_0^\infty \xi(L,\Omega)|1_\Omega, 1_{-\Omega}\rangle d\Omega$, where $|1_\Omega, 1_{-\Omega}\rangle = a^\dagger(\Omega)a^\dagger(-\Omega)|0\rangle$ and $a^\dagger(\Omega)$ is the creation operator. $\xi(L,\Omega)$ represents the two-photon amplitude of the scalar SFWM process of TE_0 mode in the Silicon waveguide. Ω is the frequency difference between the signal photon/idler photon and the pump light.

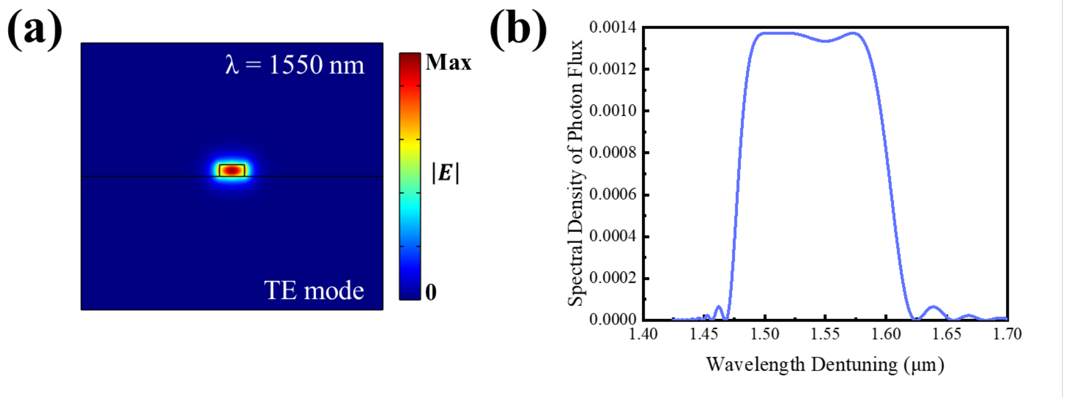

Fig. S14. The spectral density of photon flux of the Si waveguide. (a). The steady-state distribution of the electric field of TE mode for the Silicon waveguide. (b). The spectral density of

photon flux generated in the Silicon waveguide with the wavelength of the pump light is 1550 nm.

By simplifying, we can get the spectral density of photon flux $f(L,\Omega)=\frac{(\gamma PL)^2}{2\pi}\sin c^2[(\beta_2\Omega^2+2\gamma P)L/2]$. Here, the nonlinear coefficient is $\gamma=\frac{2\pi n_2}{\lambda A_{eff}}$. n_2 is the nonlinear refractive index of Silicon, and A_{eff} is the effective mode volume of the propagation mode. As shown in Fig. S14(b), we plot the spectral density of photon flux when the wavelength of pump light is 1550 nm. The theoretical result indicates that in the whole C band, the SFWM-based photon pair can be generated with a relatively high efficiency.

Additionally, we would like to point out that the use of the ring resonator might bring the effect of the nonlinear dispersion. In this situation, the signal and idler photons at the FSRs far from the pump optical frequency may not meet the energy conservation required by the SFWM process. To avoid this phenomenon, we increase the coupling strength in the design, resulting in a reduction of the Q factor and an expansion of the resonant region.

2. In the experiment, we also measure the photon pair generation of our hyperbolic quantum source in the wavelength range from 1535 nm to 1567 nm, when the wavelength pump light is set as 1550.92 nm (193.3 THz), as shown in Fig. S15. The pump power is set as 2.2 mW (in the waveguide). In this measurement, the frequency range is over 6 FSRs of our hyperbolic quantum source. We can see that, in every FSR, the high-brightness photon-pair source can be realized.

On the other hand, the corresponding CARs are also measured. We find that the generated photon pairs in the two FSRs near the pump light wavelength have very low CARs. The main reason is that the frequency interval between the pump photons and signal (idler) photons is too small, which leads to the filter system (constructed by cascaded DWDMs) not filtering out all pump photons. The pump photons that are not filtered out become a part of the noise photons. Thus, the CAR is greatly reduced.

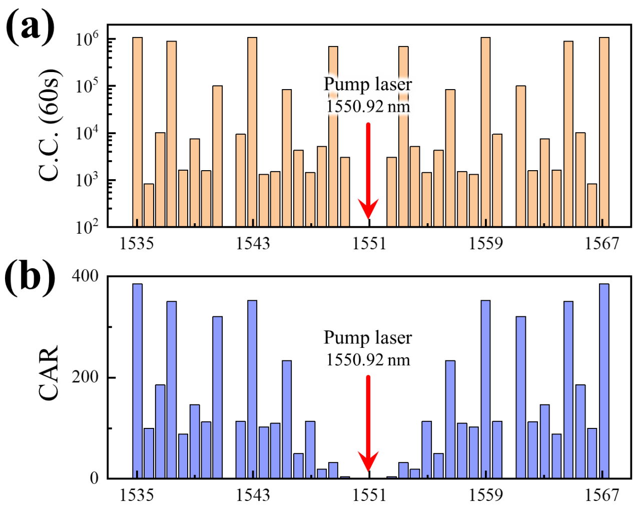

Fig. S15. The C.C. and CAR results measured in the different frequency channels. (a). The coincidence counts in 60s at the output port (adjusted for coupling losses), with different frequency channels of the signal and idler photons. (b). The corresponding CAR of the generated signal and idler photons in different frequency channels.

Based on the above theoretical and experimental results, we can conclude that there is no difference in brightness even if the signal and idler photons are significantly further separated in frequency. But, For the CAR, we can obtain a high-CAR photon pair by using the frequency channels further away from the frequency of the pump light.

So, in order to obtain the high-CAR results, we have updated the experimental results in this work, by using the frequency channels of signal and idler photons being +3 FSR and -3 FSR.

S7. The discussion of the JSI for the distinguishable and indistinguishable photons.

S7.1. The JSI for the distinguishable photons.

Here, we introduce the experimental set-up to measure the JSI for the distinguishable photons. As shown in Fig. S16(a), the pump set-up includes the laser, the filter system 1 (FS1), and the polarization controllers (PCs). The single pump laser with the frequency being 193.3 THz (C33) goes the FS1 and is injected into the hyperbolic structure. Fig. S16(b) shows that the FS1 is constructed by single DWDM, whose filter bandwidth is 100 GHz, and the central frequencies are 193.3 THz (C33). Fig. S16(d) plots the transmission of this C33 DWDM. We can see that the extinction ratios are more than 70 dB and the insertion losses are about 2dB, which shows a good performance of the filter.

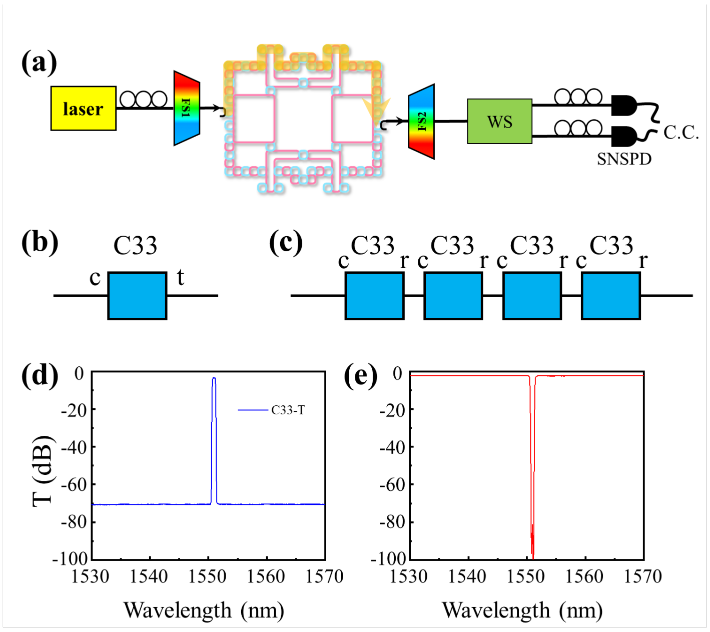

Fig. S16. The experimental set-up to measure the JSI for the distinguishable photons. (a). The experimental set-up. (b). The construction of the FS1. (c). The construction of the FS2. (d). The transmission of the FS1. (e). The transmission of the FS2.

After going through the hyperbolic structure, the pump light and the generated photons will enter into the filter system 2 (FS2), which is employed to filter out the pump photons. As shown in Fig. S16(c), we use four C33 DWDMs to carry out this function of the band elimination filter. Fig. S16(e) plots the transmission of the FS2. We can see that the extinction ratios are about 100 dB and the insertion losses are about 3 dB, which shows a good performance.

And then, the generated signal and idler photons are injected the 1×4 ports waveshaper (Waveshaper 4000A) and two SNSPDs to measure the JSI, which is shown in Fig. 2g in the main text.

S7.2. The JSI for the indistinguishable photons.

Here, we introduce the experimental set-up to measure the JSI for the indistinguishable photons. As shown in Fig. S17(a), the pump set-up includes the laser, the filter system 1 (FS1), and the polarization controllers (PCs). The two pump lasers with the frequencies being 192.3 THz (C23) and 194.3 THz (C43) are combined by the FS1 and injected into the hyperbolic structure. Fig. S17(b) shows that the FS1 is constructed by two DWDMs, whose filter bandwidth is 100 GHz, and the central frequencies are 192.3 THz (C23) and 194.3 THz (C43). Fig. S17(d) plots the transmission of these two DWDMs. We can see that the extinction ratios are more than 70 dB and the insertion losses are about 2dB, which shows a good performance of the filters.

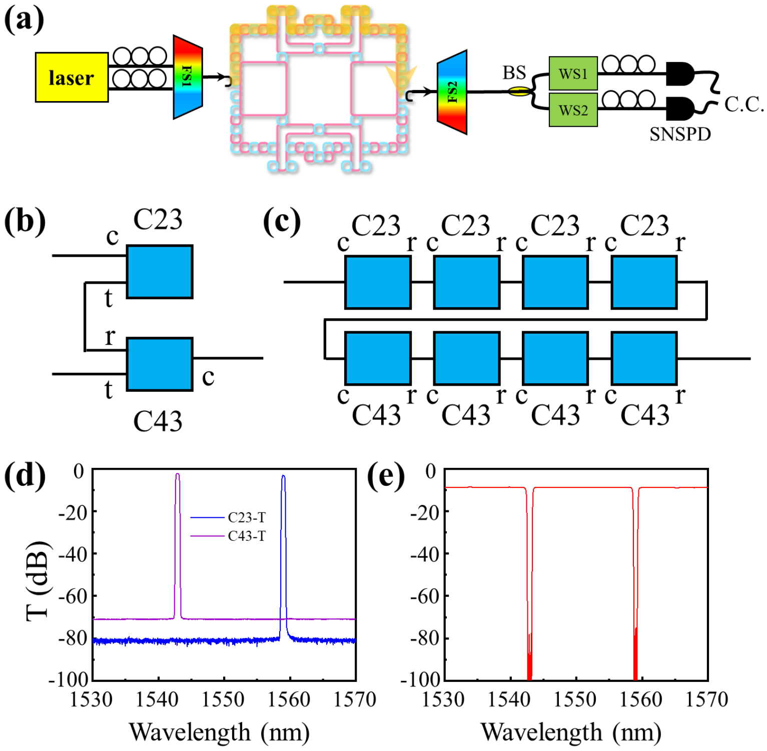

Fig. S17. The experimental set-up to measure the JSI for the indistinguishable photons. (a). The experimental set-up. (b). The construction of the FS1. (c). The construction of the FS2. (d). The transmission of the FS1. (e). The transmission of the FS2.

After going through the hyperbolic structure, the pump light and the generated photons will enter into the filter system 2 (FS2), which is employed to filter out the pump photons. As shown in Fig. S17(c), we use eight DWDMs (four C23 and four C43) to carry out this function of the band elimination filter. Fig. S17(e) plots the transmission of the FS2. We can see that the extinction ratios are about 100 dB and the insertion losses are about 9 dB, which shows a good performance.

And then, the generated signal and idler photons are injected in a 50:50 beam splitter (BS). There are signal (idler) photons existing in each path. We use two waveshapers (Waveshaper 4000A)

and two SNSPDs to measure the JSI, which is shown in Fig. 4c in the main text.

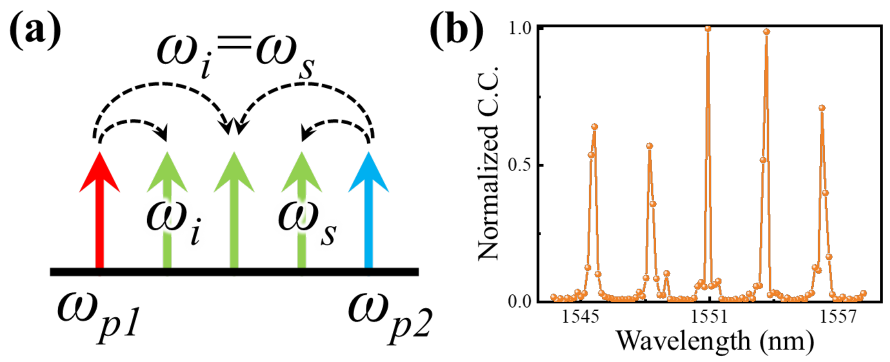

Fig. S18. The dual-pump SFWM process. (a). The theorical scheme of the two-wavelength pumping SFWM process. (b). Normalized C.C. in the diagonal line of the JSI image in Fig. 4c of the main text.

It is worth pointing out that there are not only the indistinguishable photons (with same frequencies) but also the distinguishable photons (with different frequencies) generated in this process. The theorical scheme is shown in Fig. S18(a). Thus, we also measure the additional experimental data in the diagonal line of the JSI image in Fig. 4c of the main text, as shown in Fig. S18(b). It means that, in this measurement, we set the wavelength of the signal photon from 1540 nm to 1560 nm and the corresponding wavelength of the idler photon from 1560 nm to 1540 nm. And the sum of the wavelengths of the signal and idler photons remains unchanged (being the sum of the two pump wavelengths, 1558.98 nm + 1542.94 nm). The experimental results indicate that the distinguishable photons are also generated in the SFWM process under the two-wavelength pumping.

S8. Simulation demonstration of topological protection of the hyperbolic topological insulator.

In this part, we present numerical results on robust edge propagations in the hyperbolic photonic topological insulator. The defective hyperbolic photonic topological insulator is shown in Fig. S19(a). It is shown that the input light can propagate along the upper and lower edge channels, which are corresponds to the topological spin-up and spin-down modes. Especially, the light propagating along the upper edge channel will meet the defect, while the light propagating along the lower edge channel cannot meet the defect. Using the finite element method, we calculate the transmission spectra of topological photonic topological insulators, as shown in Figs. S19(b). The simulation results show the calculated transmission spectra under the excitation of the topological spin-up mode. The shaded area corresponds to the region of topological edge modes. By comparing this result with that of Fig. S11, we can see that the transmission spectra are almost identical, indicating that the structure can support topologically protected edge modes.

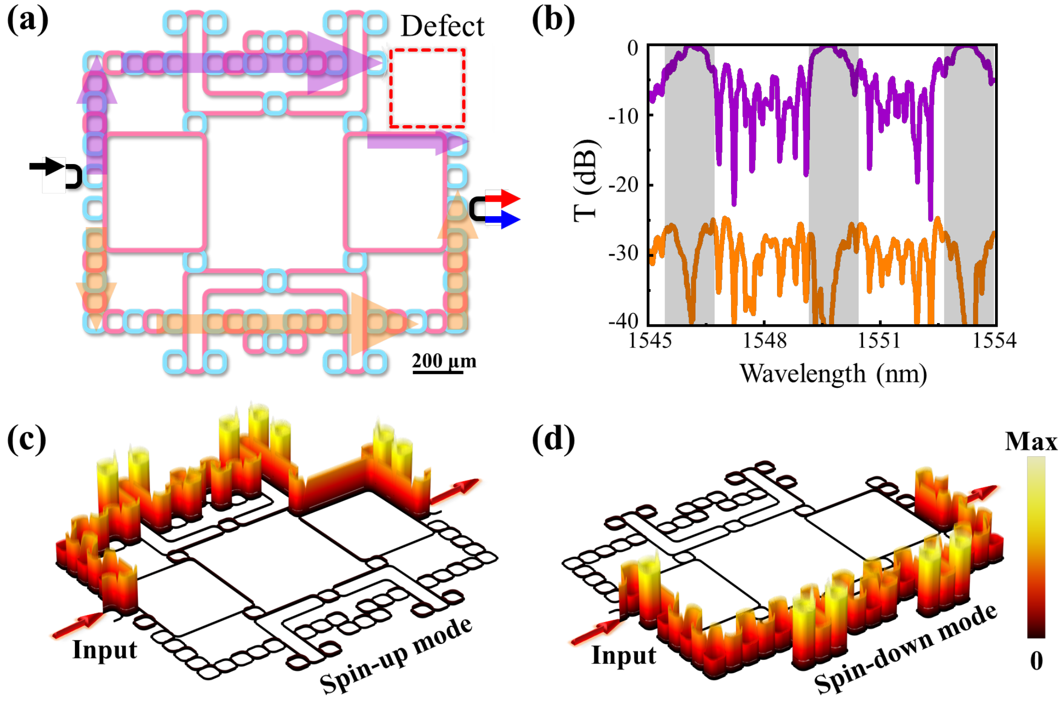

Fig. S19. The simulation results of the hyperbolic topological structure with the defect. (a) The schematic diagram of the hyperbolic topological photon structure with the defect, with a certain defect located in the upper right corner. (b) Simulated transmission spectra by exciting the spin-up mode. Simulation results of steady-state distributions of electric fields by exciting (c) the topological spin-up mode and (d) the topological spin-down mode with the wavelength being 1549.7 nm.

In order to intuitively display the topological one-way propagation, Figs. S19(c) and S19(d) present the steady-state distributions of electric fields by exciting the spin-up and spin-down modes at 1549.7 nm. It also shows the one-way transport behavior of input lights even though the defect exists. By the comparison with the simulation shown in Fig. S11, we can conclude that the topological protection of our designed hyperbolic topological insulators is demonstrated.

S9. The robustness comparison of the hyperbolic lattice, the square Euclidean lattice, and the rectangular Euclidean lattice.

Here, we provide a robustness comparison of the hyperbolic lattice, the square Euclidean lattice, and the rectangular Euclidean lattice. As shown in Fig. S20(a), the 2-layer hyperbolic lattice is constructed. The total number of the sites is 48, and the edge (bulk) site number is 42 (6). For comparison, we also construct the square and rectangular Euclidean lattices with the same number of sites, as shown in Figs. S20(b)-S20(d). Next, we present numerical results on the robust edge propagations of these models.

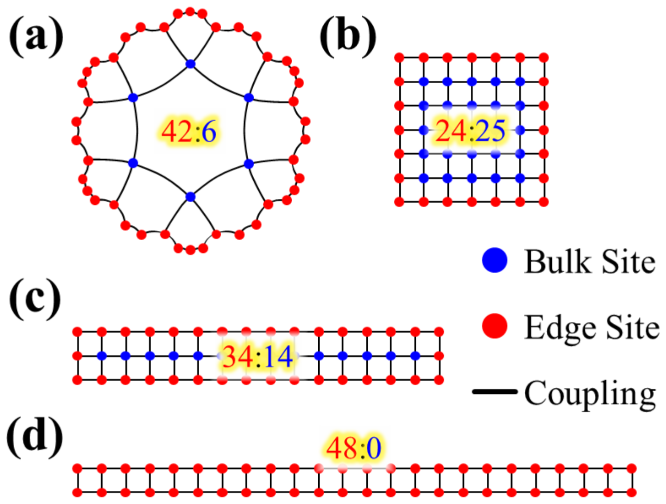

Fig. S20. The illustration of the hyperbolic and Euclidean topological insulator. (a). The 2-layer hyperbolic lattice. The total number of sites is 48. The rate between the edge and bulk sites is 42:6. (b). The square Euclidean lattice. The total number of sites is 49 (7 by 7). The rate between the edge and bulk sites is 24:25. (c). The rectangular Euclidean lattice with bulk site. The total number of sites is 48 (16 by 3). The rate between the edge and bulk sites is 34:14. (d). The rectangular Euclidean lattice without bulk site. The total number of sites is 48 (24 by 2). The rate between the edge and bulk sites is 48:0. Here, the red dots represent the edge sites. Blue dots represent the bulk sites. Black lines represent the coupling.

Firstly, we calculate the transmission spectra of the propagation mode without the disorder. The calculation results are plotted in the black lines in Figs. S21(a)-S21(d). And then, for quantifying the robustness, we introduce the random disorder θ in every site. The disorder is set as a random variation in ring resonance frequencies. The maximum of random disorder is set to 0.6J, 0.8J, 1.2J, and 1.6J, respectively. After 100 times calculations, the numerical simulation results of these four models show the different degrees of topological robustness, as shown in Figs. S21(a)-S21(d).

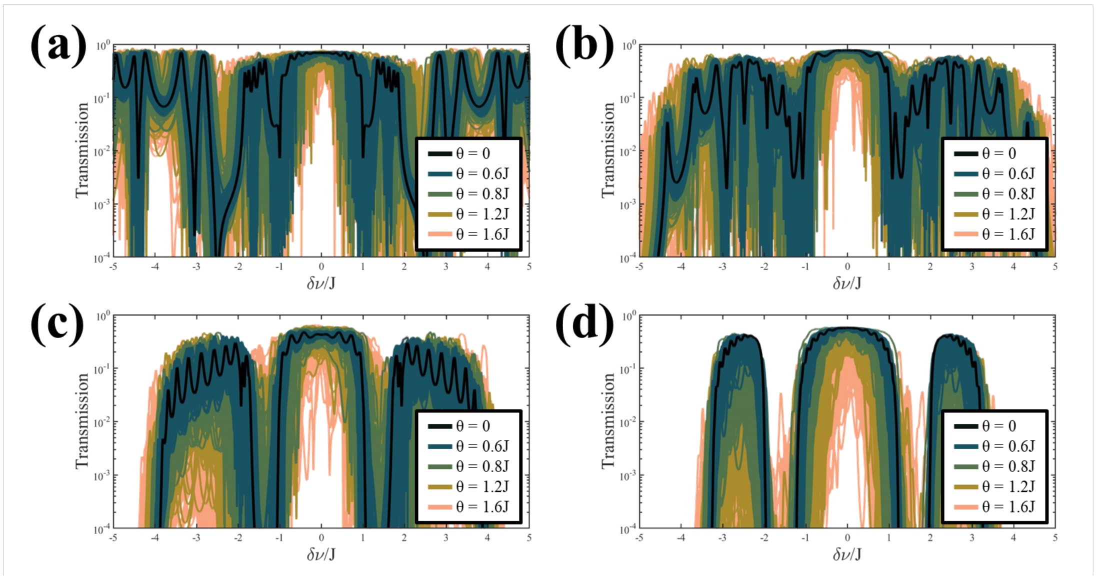

Fig. S21. Topological robustness of edge modes of hyperbolic and Euclidean lattices against random variations in ring resonance frequencies, for (a) the 2-layer hyperbolic lattice, (b) the 7 by 7 square Euclidean lattice, (c) the 16 by 3 rectangular Euclidean lattice, (d) the 24 by 2 rectangular Euclidean lattice, respectively. Here, $\delta v = f - f_0$, where f is the excitation frequency, f_0 is the ring resonance frequency without the disorder.

It can be seen that the edge states exist in the frequency range from -1J to +1J, and the bulk modes exist in other frequency ranges. After introducing the disorders, the edge states can also remain relatively high transmissions. The bulk modes are drastically affected and cannot remain a high transmission. Now, we focus on the edge state, and quantitatively study the topological robustness of the hyperbolic lattice, the square Euclidean lattice, and the rectangular Euclidean lattice.

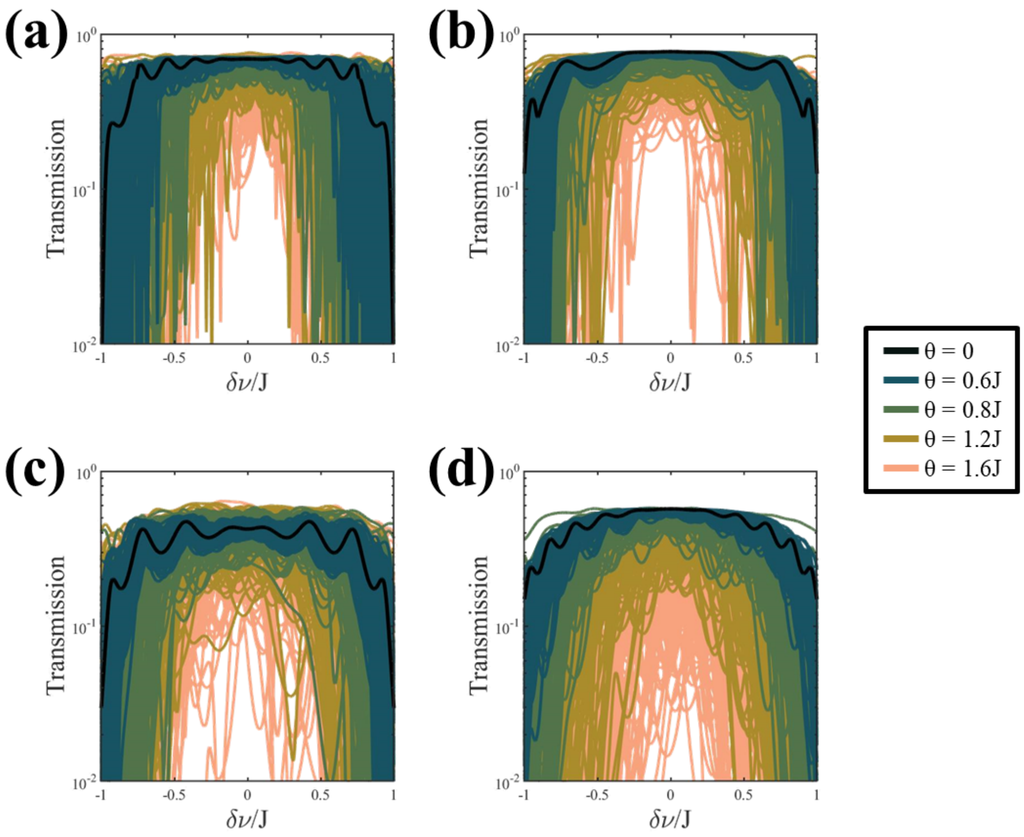

Fig. S22. The enlarged figures of Fig. S21. (a)-(d) correspond to Fig. S21(a)-Fig. S21(d), respectively. These enlarged figures focus on the edge mode.

We plot the enlarged figures of Fig. S21 to clearly show the influence to the edge state from the disorders, as shown in Fig. S22. It can be seen that the transmission of the edge state does not decrease much in the hyperbolic lattice and the square Euclidean lattice, as shown in Figs. S22(a) and S22(b). As for the rectangular Euclidean lattices, the effect from the random disorders is more dramatic. Such a phenomenon proves that the hyperbolic topological lattice has the same degree of topological robustness as the square Euclidean lattice. By changing the square Euclidean topological lattice to the rectangular Euclidean topological lattice, the ratio between edge sites and bulk sites can indeed be increased, but the topological robustness will be weakened.

Additionally, we also calculate the energy spectra of the eigen-mode without the disorder. The calculation results have been plotted in the black lines in Figs. S22a-S22b. And then, for quantifying the robustness, we introduce the random disorder θ in every site. The disorder is set as a random variation in ring resonance frequencies. The maximum of random disorder is set to 0.6J, 0.8J, 1.2J, and 1.6J, respectively. After 100 times calculations, the numerical simulation results of these four models show the different degrees of topological robustness, as shown in Figs. S22a- S22b.

We have calculated the eigenenergy spectra, topological invariants (Chern numbers), and phase distribution diagrams for Euclidean and hyperbolic topological insulators under random disorder magnitudes of 0J, 1J, 2J, and 3J. The results are shown in Fig. S23, where panels Figs. S23a-S23d correspond to the Euclidean topological insulator, and panels Figs. S23e-S23h correspond to the hyperbolic topological insulator. The four rows from top to bottom represent the cases with

increasing disorder magnitudes from 0J to 3J, respectively.

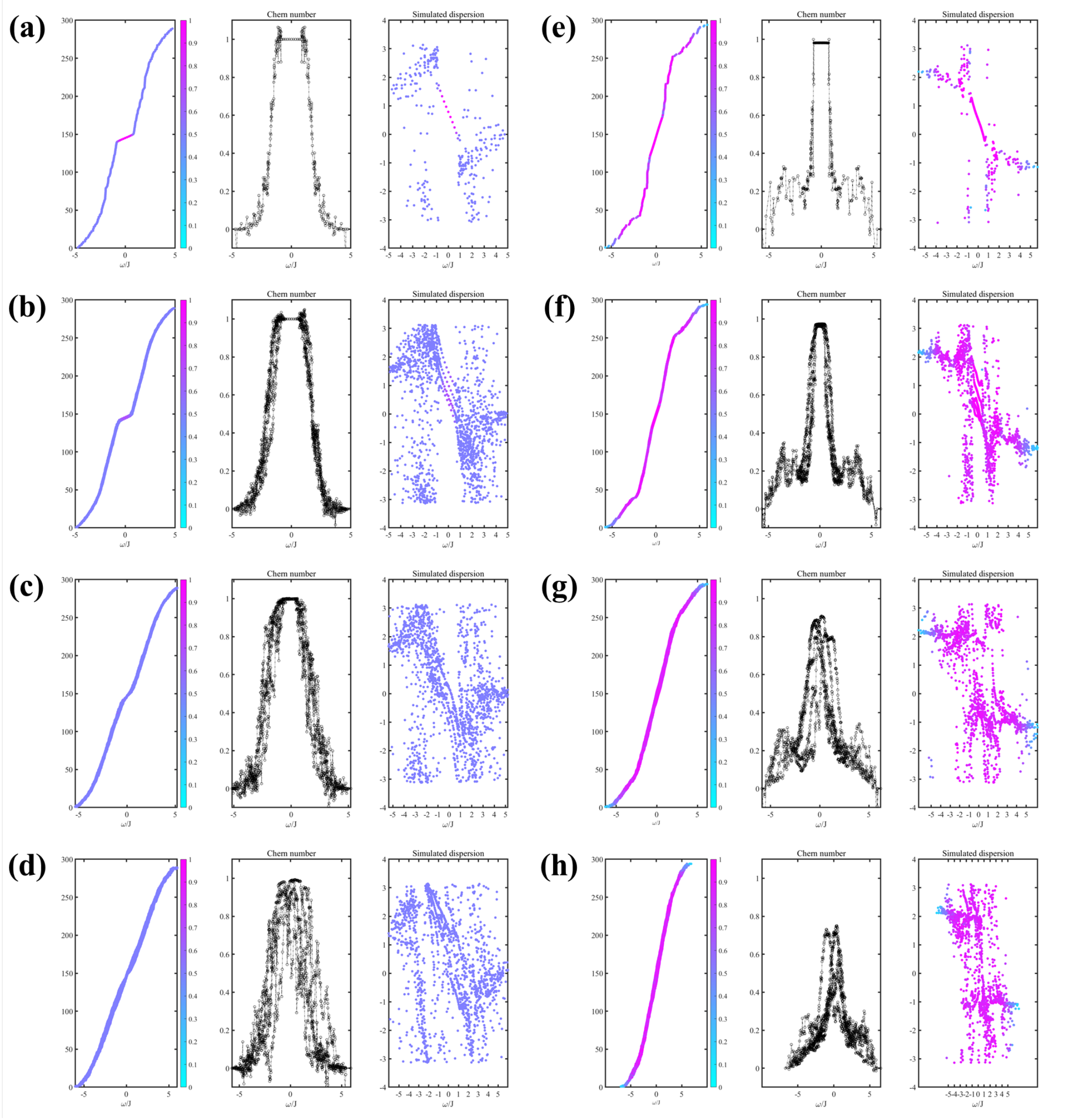

Fig. S23. Quantitative study of topological protection in Euclidean and hyperbolic lattices. Panels (a)-(d) correspond to the Euclidean topological insulator, while panels (e)-(h) correspond to the hyperbolic topological insulator. In each panel, the eigenenergy spectra, topological invariants (Chern numbers), and phase distribution diagrams are displayed in the three sections from left to right, respectively. The four rows from top to bottom represent the cases with increasing disorder magnitudes of 0J, 1J, 2J, and 3J, respectively.

We can observe that the topological bandgap of the Euclidean lattice is slightly larger than that of the hyperbolic lattice, as shown in Figs. S23a and S23e. However, the platforms with Chern numbers being 1 are both centered in the energy spectra for both lattices. As the random disorder increases, their bandgaps gradually close, primarily manifested by the gradual disruption of the Chern number platforms, the disappearance of edge states, and the loss of linear phase response. Specifically, we find that for the Euclidean lattice, the bandgap completely closes when the random disorder exceeds 3J, while for the hyperbolic lattice, the bandgap closes when the random disorder exceeds 2J. Therefore, we can quantitatively state that both hyperbolic and Euclidean lattices exhibit

a certain degree of topological protection against random disorders. And, the Euclidean lattice demonstrates slightly stronger topological robustness compared to the hyperbolic lattice.

S10. The 10 times measurements and a long-coincidence-counting-time measurement of the energy-time entanglement curves.

Here, we perform 10 times measurements and a long-coincidence-counting-time measurement of the energy-time entanglement curves. These experimental results are shown in Fig. S24 and Fig. S25. By the fitting, we also obtain the corresponding visibilities, as shown in Table. S1.

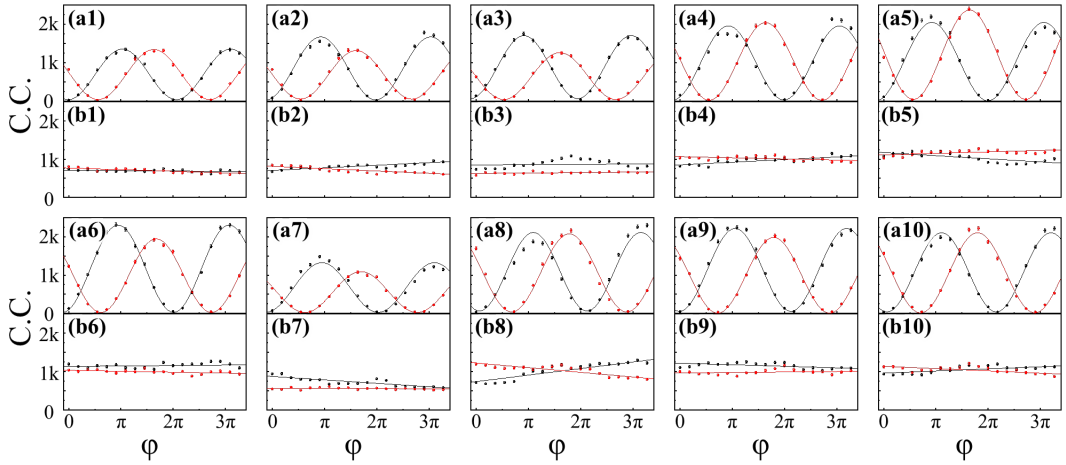

Fig. S24. The 10 times measurements of two-photon interferences under two nonorthogonal bases. (a1)-(a10) correspond to the C.C. of the central peak. (b1)-(b10) correspond to the sum of the C.C. of left and right peaks. The error bars are calculated assuming Poissonian statistics for photon counts. The coincidence counting time is 30 s.

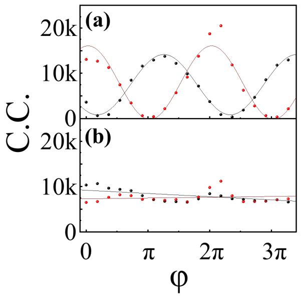

Fig. S25. The long-coincidence-counting-time measurement of two-photon interferences under two nonorthogonal bases. (a) corresponds to the C.C. of the central peak. (b) correspond to the sum of the C.C. of left and right peaks. The error bars are calculated assuming Poissonian statistics for photon counts. The coincidence counting time is 5 min.

Table. S1. The visibilities of measured interference curves for the energy-time entangled photon pair

No.	Coincidence counting time	Visibility of black curve	Visibility of red curve
1	30 s	96.46±0.64%	96.79±0.62%
2	30 s	98.01±0.47%	96.12±0.68%
3	30 s	95.72±0.69%	95.64±0.74%
4	30 s	96.19±0.58%	96.84±0.46%
5	30 s	97.96±0.43%	97.18±0.43%
6	30 s	95.52±0.59%	94.13±0.54%
7	30 s	94.91±0.80%	94.20±0.77%
8	30 s	97.33±0.47%	96.02±0.51%
9	30 s	96.31±0.48%	97.20±0.49%
10	30 s	97.33±0.41%	97.04±0.45%
11	5 min	95.40±0.25%	96.77±0.17%

We can see that these experimental results indicate that our hyperbolic topological quantum source can generate two energy-time entangled photons. All the experimental results prove that our hyperbolic topological quantum source can generate two energy-time entangled photons with high visibilities.

S11. The connection type of the FS1 and FS2.

Here, let us introduce the connection types of the FS1 and FS2 of Fig. 4(a) in the main text. We firstly consider a three-port DWDM, whose ports are named as c, t, and r, as shown in Fig. S26(a). If we inject a light with continuous spectrum into the port c, only the partial light with special frequency can be transmitted to the port t, and the rest light goes to the port r. That is to say, the device can be seen as a combination of a band-pass filter (from c port to t port) and a band elimination filter (from c port to r port). We can also measure their experimental transmissions, as plotted in blue and red lines of Fig. S26(b), corresponding to the cases from c to t and from c to r, respectively. This result shows a large extinction ratio (more than 60 dB), which can effectively reduce the influence of noise photons.

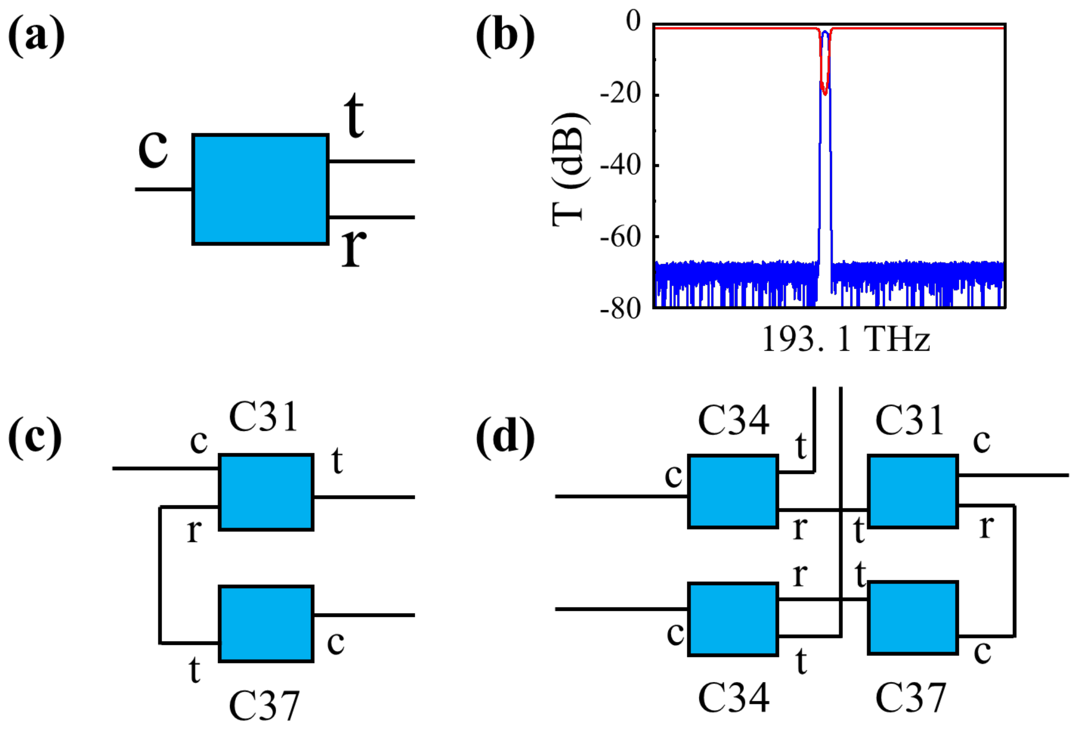

Fig. S26. The dense wavelength division multiplexer and filter system. (a) The schematic diagram of the DWDM. (b) The transmission spectrum of the DWDM. The blue line represents the transmission from c port to t port. The red line represents the transmission from c port to r port. The schematic diagrams of the connection types of (c) the FS1 and (d) the FS2. Here, C31, C34, and C37 represents the filtering frequency being 193.1 THz, 193.4 THz, and 193.7 THz.

And then, we use two three-port DWDMs to construct the FS1, as shown in Fig. S26(c). In this way, the FS1 can combine two lights with frequencies of 193.1 THz and 193.7 THz from two right ports and output them to the left port. As for the FS2, as shown in Fig. S26(d), four three-port DWDMs are employed to realize its function. To be specific, the mixed light is firstly filtered into two left output ports. The light of the frequency being 193.7 THz (193.1 THz) went to the left-upper (left-lower) port. Additionally, after going through the Sagnac interferometer, the pump lights and the generated photons (193.4 THz) came back to the left ports of the FS2. Finally, the FS2 could also filter out generated photons and make them enter into the detection set-up.

Reference

S1. K. Ikeda, S. Aoki, and Y. Matsuki, Hyperbolic band theory under magnetic field and Dirac cones on a higher genus surface. J. Phys. 33, 485602 (2021).

S2. D. Leykam, S. Mittal, M. Hafezi, and Y. D. Chong, Reconfigurable topological phases in next-nearest-neighbor coupled resonator lattices. Phys. Rev. Lett. 121, 023901 (2018).

S3. E. Brainis, Four-photon scattering in birefringent fibers. Phys. Rev. A 79, 023840 (2009).

S4. A. Biberman, M. J. Shaw, E. Timurdogan, J. B. Wright, and M. R. Watts, Ultralow-loss silicon ring resonators. Opt. Lett. 37, 4236-4238 (2012).

S5. X. Ji, F. A. S. Barbosa, S. P. Roberts, A. Dutt, J. Cardenas, Y. Okawachi, A. Bryant, A. L. Gaeta, and M. Lipson, Ultra-low-loss on-chip resonators with sub-milliwatt parametric oscillation threshold. Optica 4, 619-624 (2017).
